# Blood immune cells as potential biomarkers predicting relapse-free survival of stage III/IV resected melanoma patients treated with peptide-based vaccination and interferon-alpha

**DOI:** 10.3389/fonc.2023.1145667

**Published:** 2023-05-18

**Authors:** Federica Moschella, Carla Buccione, Irene Ruspantini, Luciano Castiello, Andrea Rozo Gonzalez, Floriana Iacobone, Virginia Ferraresi, Belinda Palermo, Paola Nisticò, Filippo Belardelli, Enrico Proietti, Iole Macchia, Francesca Urbani

**Affiliations:** ^1^ Department of Oncology and Molecular Medicine, Istituto Superiore di Sanità, Rome, Italy; ^2^ Core Facilities, Istituto Superiore di Sanità, Rome, Italy; ^3^ Department of Medical Oncology 1, Scientific Institute for Research, Hospitalization and Healthcare (IRCCS) Regina Elena National Cancer Institute, Rome, Italy; ^4^ Tumor Immunology and Immunotherapy Unit, Department of Research, Advanced Diagnostics and Technological Innovation, Scientific Institute for Research, Hospitalization and Healthcare (IRCCS) Regina Elena National Cancer Institute, Rome, Italy; ^5^ Institute of Translational Pharmacology, National Research Council (CNR), Rome, Italy; ^6^ Medical Biotechnology and Translational Medicine PhD School, II University of Rome “Tor Vergata”, Rome, Italy

**Keywords:** immunotherapy, melanoma, combination therapy, multiparametric flow cytometry, circulating biomarkers, peptide vaccination, interferon alpha (IFN-α), adjuvant therapy

## Abstract

**Introduction:**

Despite the recent approval of several therapies in the adjuvant setting of melanoma, tumor relapse still occurs in a significant number of completely resected stage III-IV patients. In this context, the use of cancer vaccines is still relevant and may increase the response to immune checkpoint inhibitors. We previously demonstrated safety, immunogenicity and preliminary evidence of clinical efficacy in stage III/IV resected melanoma patients subjected to a combination therapy based on peptide vaccination together with intermittent low-dose interferon-α2b, with or without dacarbazine preconditioning (https://www.clinicaltrialsregister.eu/ctr-search/search, identifier: 2008-008211-26). In this setting, we then focused on pre-treatment patient immune status to highlight possible factors associated with clinical outcome.

**Methods:**

Multiparametric flow cytometry was used to identify baseline immune profiles in patients’ peripheral blood mononuclear cells and correlation with the patient clinical outcome. Receiver operating characteristic curve, Kaplan-Meier survival and principal component analyses were used to evaluate the predictive power of the identified markers.

**Results:**

We identified 12 different circulating T and NK cell subsets with significant (p ≤ 0.05) differential baseline levels in patients who later relapsed with respect to patients who remained free of disease. All 12 parameters showed a good prognostic accuracy (AUC>0.7, p ≤ 0.05) and 11 of them significantly predicted the relapse-free survival. Remarkably, 3 classifiers also predicted the overall survival. Focusing on immune cell subsets that can be analyzed through simple surface staining, three subsets were identified, namely regulatory T cells, CD56^dim^CD16^-^ NK cells and central memory γδ T cells. Each subset showed an AUC>0.8 and principal component analysis significantly grouped relapsing and non-relapsing patients (p=0.034). These three subsets were used to calculate a combination score that was able to perfectly distinguish relapsing and non-relapsing patients (AUC=1; p=0). Noticeably, patients with a combined score ≥2 demonstrated a strong advantage in both relapse-free (p=0.002) and overall (p=0.011) survival as compared to patients with a score <2.

**Discussion:**

Predictive markers may be used to guide patient selection for personalized therapies and/or improve follow-up strategies. This study provides preliminary evidence on the identification of peripheral blood immune biomarkers potentially capable of predicting the clinical response to combined vaccine-based adjuvant therapies in melanoma.

## Introduction

Following complete surgical resection, stage III-IV melanoma patients have high-risk of recurrence (1, 2). The evolving landscape of adjuvant therapies approved for this setting of patients includes immune checkpoint inhibitor-based immunotherapy and targeted therapy (3, 4). The relapse-free survival (RFS) rates was shown to be 40.8% (at 5 years) with ipilimumab (5, 6), 51.7% (at 4 years) with nivolumab and 63.7% (at 3 years) with pembrolizumab (7–10). While Ipilimumab showed high rates (54.1%) of severe adverse events, nivolumab and pembrolizumab were better tolerated (14.4% and 14.7%) (7–10). Treatment with dabrafenib plus trametinib of stage III patients with BRAF^V600^ mutation resulted in 4-year RFS rates of 54%, associated to 41% of grade 3-4 adverse events (11, 12). Despite the unprecedented effectiveness of these novel adjuvant therapies, a significant number of stage III-IV patients still show tumor relapse due to multiple mechanisms of therapy resistance, highlighting the need of innovative treatments for melanoma recurrence prevention (13).

We previously carried out a phase I/II clinical trial and an open-label, randomized, phase II trial to determine the safety, immunogenicity and preliminary clinical efficacy of peptide-based vaccination in combination with low-dose non continuous interferon (IFN)-α2b, with or without dacarbazine preconditioning (1 day before vaccination), in patients with resected stage III, IVM1a and IVM1b melanoma (14, 15).

As a follow-up to the demonstration of safety and immunogenicity of the double and triple combination therapy in the phase I/II study (14), 34 completely resected patients were vaccinated with 2 peptides (Melan-A/MART-1 and NY-ESO-1), emulsified with Montanide ISA-51, administered in combination with 6 MU IFN-α2b (6 cycles of 2 vaccine doses), with (arm 2) or without (arm 1) dacarbazine preconditioning (15). The 4.5 years RFS rates were 52.9% and 35.3% in arm 1 and 2, respectively. The 4.5-year OS rates were 68.8% and 62.7% in arm 1 and 2, respectively. No significant differences were observed between the two arms for both RFS and OS. Our treatment was very well tolerated, with grade 3 adverse events observed in only 5.9% of patients, and absence of grade 4 adverse events. Immunological studies showed that both treatments induced a significant expansion of vaccine-specific CD8^+^ T cells, with no correlation with the clinical outcome. However, the increase of polyfunctionality and of interleukin 2 (IL-2) production by Melan-A-specific CD8^+^ T cells, as well as the expansion/activation of natural killer (NK) cells correlated with survival (15).

The choice of this combination therapy was based on our preclinical and clinical studies showing that the antitumor efficacy of immunotherapy can be augmented by certain chemotherapeutic drugs and/or by type I IFN administration (16–20). Preconditioning with cyclophosphamide or dacarbazine may potentiate the efficacy of active and adoptive immunotherapy through several immune-mediated mechanisms (17, 21, 22), including the induction of a type I IFN gene signature, demonstrated in both animal models and cancer patients (14, 23, 24). On the other hand, IFN-α, a cytokine with pleiotropic effects on development/activation of dendritic cells (25, 26), T helper type I (Th1) cell differentiation, T cell memory turn over and NK cell activation (16, 27), has been used by our group in a few clinical studies as a vaccine adjuvant (27). In stage IV advanced melanoma patients, we showed that peptide-based vaccination combined with low dose IFN-α resulted in enhanced specific CD8^+^ T cells and monocyte/dendritic cell precursor activation (28). In the same patient setting, the intratumoral injection of monocyte-derived dendritic cells generated in the presence of IFN-α, preceded by dacarbazine preconditioning, has been shown to be safe and immunogenic (29).

In all our trials, the patient selection was never based on potentially predictive biomarkers. However, emerging clinical evidence shows that, in order to improve treatment efficacy and to design potentially successful phase III trial, the use of robust predictive markers is critical. Several tissue and blood biomarkers have been revealed to predict prognosis as well as treatment response in cancer patients, enabling a more targeted application of the right therapy to the patients most likely to be treated (30–32).

Some immunological parameters, such as the frequency of certain immune cell subsets, their phenotype, their activation status and the serum concentration of some cytokines, may be used as prognostic and/or predictive markers, especially in the context of cancer immunotherapy (33, 34).

In the last few years, several papers highlighted the importance of monitoring both innate and adaptive immunity in peripheral blood to evaluate dysfunctions in cancer patients and to find relevant prognostic/predictive biomarkers for cancer immunotherapy that might facilitate patient selection and treatment decisions (32, 35). In fact, an altered peripheral blood composition may reflect altered immune responses within the tumor microenvironment. Moreover, the phenotype and function of circulating immune cell subsets may differ among individual patients with metastatic disease, influencing their response to immunotherapy. In addition, due to the low invasiveness of venous sampling, peripheral blood represents an interesting, easily accessible material to measure functional competence of immune cell subsets and to determine a “peripheral immunoscore” as previously demonstrated (36).

Here, we used multi-parametric flow cytometry to evaluate the predictive power of pretreatment circulating immune cell subsets in forecasting the clinical outcome of the melanoma patients enrolled in the above described phase II study (15), with the aim of identifying one or more potentially valuable biomarkers driving selection of patients with more chances to benefit from therapy and/or improving patient follow-up management.

## Materials and methods

### Patient enrolment, treatment and follow-up

Thirty-four HLA-A*0201, stage III/IV melanoma patients were enrolled in a single-center, open-label, randomized phase II study after tumor resection (EudraCT number 2008-008211-26). The clinical-pathological characteristics of the patient cohort, the study design, the primary and secondary objectives, the inclusion/exclusion criteria, the randomization method, the treatment and clinical follow-up are detailed in (15). Briefly, 17 patients (arm 1) received and intradermal injection of Melan-A/MART-1_26-35_ (A27L) (ELAGIGILTV) and NY-ESO-1_157-165_ (SLLMWITQC) GMP-grade peptides (Polypeptide Laboratories, Strasbourg, France) emulsified with Montanide ISA-51 (Seppic, Italy) in combination with subcutaneous injection of 6 MU IFN-α2b (IntronA^®^, Schering-Plough, USA). The immunization regimen for patients in arm 1 (n=17) consisted of 6 cycles (every 21 days) of two vaccine doses (7 days apart). Patients in arm 2 (n=17) received the same treatment of arm 1, preceded (1 day before each vaccination cycle) by an intravenous infusion of 800 mg/m^2^ dacarbazine (Deticene by Sanofi-Aventis, France).

### Immune response monitoring

A multicolor flow-cytometry-based approach was used to assess frequency, phenotype and functionality of the major circulating lymphocyte subpopulations and NK cells, before (pre), during (T92) and after treatment (4 and 6 months after enrollment).

Peripheral blood mononuclear cells (PBMCs) were isolated by Ficoll gradient (Lymphoprep Axis-Shield, Scotland) and frozen as described elsewhere (29). Samples taken at different time points were tested within the same experimental session, in accordance to “minimal information about T cell (MIATA) and NK (MIANKA) assays” guidelines (http://miataproject.org/miata-guidelines/final-guidelines-2/), to improve the data quality level of flow cytometry assay. Cells were thawed in the presence of DNase. Live and dead cells were discriminated by trypan blue exclusion method and samples showing viability less than 70% were not further processed.

### Flow cytometry panels

The antibodies (Abs) used in each panel are described in **Supplementary Table S1**.

#### Total and CD4/CD8/γδ T cell subpopulations associated with memory-naïve phenotype

Briefly, to quantify the major T lymphocyte subsets, PBMCs were stained with a six-color panel consisting of a mixture of the following monoclonal antibodies (mAbs): anti-Vδ2, anti-CD3, anti-CD4, anti-CD8, anti-CD45RA and anti-CCR7 mAbs. The gating strategies for the different T cell subsets are depicted in **Supplementary Figure S1**. In particular, within the lymphocyte region (**Figure S1A**), using a CD3 vs. Vδ2 plot, we identified CD3^+^ γδ^+^ T cells, as well as total CD3^+^ and CD3^-^ cells (**Figure S1B**, left plot). The CD3^+^ T cells were further distinguished in CD4^+^, CD8^+^ single-positive and CD4^+^CD8^+^ double positive T cell subsets. This last subset was further divided into CD8^low^CD4^hi^ and CD8^hi^CD4^low^ T cells (**Figure S1B**, right panel) (37). The memory phenotype of different T subsets (based on the expression of CD45RA and CCR7) is shown in **Supplementary Figure S1C**.

#### Regulatory T cells

To enumerate circulating regulatory T cells (Tregs), a panel consisting of anti‐CD45, anti‐CD3, anti‐CD4, anti‐CD25, anti‐FoxP3 and anti‐CD127 conjugated mAbs was applied. Markers for Tregs have been chosen based on a consensus published paper (38). For intranuclear staining of Foxp3, we employed the eBioscience™ Foxp3/Transcription Factor Staining Buffer Set and protocol (e-Biosciences, Massachusetts), which includes fixation and permeabilization buffers. Tregs were defined as CD3^+^, CD4^+^, CD25^hi^, CD127^-^ and Foxp3^+^, as depicted in **Supplementary Figure S1D**.

#### Total CD8^+^ and Melan-A^+^ CD8^+^ T cell functionality

Functional analysis of total and vaccine-specific T cell responses was performed on cryopreserved PBMCs by a previously described functional multiparametric test (15, 39), consisting of surface staining for CD8 and HLA-A*0201/Melan-A tetramer, staining for the cytotoxicity surrogate marker CD107a and intracellular staining for IFN-γ, IL-2, and TNF-α cytokines. Briefly, 2 × 10^6^ PBMCs/well were stained with PE-labeled HLA-A*0201/Melan-A tetramer (0.5 μg/10^6^ cells), washed, and cultured in 96-well round-bottom plates in the presence of anti-CD49d and anti-CD28 costimulatory Abs (Becton Dickinson, San Jose, CA, USA), for 1 hour at 37°C in a 5% CO_2_ incubator. The RPMI medium (Life Technologies, Gibco BRL, Grand Island, NY, US) was complemented with 2% human serum (Euroclone, Pero, Italy), HEPES, penicillin, streptomycin, nonessential amino acids, L-glutamine and DNase I (10 U/mL). Staphylococcal enterotoxin B (SEB; Sigma-Aldrich, Munich, Germany) (2 μg/mL) was used as positive control. During the incubation, PBMCs were stained with FITC-labeled anti-CD107a. Brefeldin A (Golgi Plug) and monensin (Golgi stop) (Becton Dickinson) were added for additional 5 hours to prevent cytokine secretion and lysosome acidification, respectively. Cells were then incubated for 10 min at room temperature with 2 mM EDTA. Cells were surface stained with PE/Cy7-conjugated anti-CD8 mAb (30 min at 4°C) and then washed, fixed, permeabilized with BD IntraSure kit (BD Biosciences, San Jose, CA, USA) and intracellularly stained with an Ab cocktail containing fluorescently labeled mAbs directed against IFN-γ, IL-2, and TNF-α. The gating strategies for the functional analysis of total CD8^+^ T cells and Melan-A-specific CD8^+^ T cells are depicted in **Supplementary Figures S2A-C**.

#### NK cell subsets and functionality

NK cells were stained with the following Abs: anti-CD3, anti-IFN-γ, anti-CD107, anti-CD56, anti-CD16 and LIVE/DEAD Fixable Near-IR Dead Cell Stain Kit (Molecular Probes, Eugene, OR, USA). Total NK cells were phenotypically identified by CD56 positivity within the CD3 negative lymphocytes, and further discriminated into four different subsets, namely CD56^dim^CD16^+^ NK, CD56^hi^CD16^-^ NK, CD56^dim^CD16^-^ NK and CD56^hi^CD16^+^ NK (**Supplementary Figure S1D**).

NK cell functionality was determined in a single-cell assay using CD107a mobilization assay and IFN-γ production, as described in our previous paper (15). Cells were stimulated with K562 cells at 25:1 effector/target ratio or PMA (1.25 ng/mL) and ionomycin (1μg/mL) (Sigma-Aldrich, St. Louis, MO, USA), as positive control. In brief, 1 × 10^6^ thawed PBMCs were cultured in U-bottom plates for 4 h at 37°C cells in the presence of monensin (Golgi Stop; BD Biosciences) and brefeldin A (Golgi Plug; BD Biosciences). FITC-labeled anti-CD107a mAb was added at the beginning of the incubation. After culturing, cells were labeled for 20 min at 4°C with anti-CD16, anti-CD56, and anti-CD3 mAbs. Cells were then washed, lysed, and permeabilized with BD IntraSure kit (BD Biosciences) and stained with anti-IFN-γ mAb. The LIVE/DEAD Fixable Near-IR Dead Cell Stain Kit was used to determine the viability of cells prior to surface and intracellular staining. FcR blocking (BD Biosciences) was also included in order to avoid non-specific staining of mAbs to FcγRIII. Spontaneous degranulation (CD107a^+^ percentage) and IFN-γ secretion were determined in the absence of targets and stimuli. The gating strategy for functional NK test in shown in **Supplementary Figure S2E**.

### Flow cytometry analyses

Data acquisition was performed using a FACSCanto instrument (BD Biosciences, CA) and analyzed either by FACS DIVA (BD Biosciences, CA) or FlowJo v.10 (Tree Star, Ashland, OR, USA) or Kaluza v.1.3 (Beckman Coulter, Brea, CA, USA) software. In order to give statistical significance to poorly expressed or even rare cell populations, up to 1,300,000 events were acquired for each sample. Abnormal or manifestly artifact samples were excluded from analysis (e.g., light scatter or any fluorescence abnormal profile).

### Statistical analysis

The variables generated by flow cytometric analysis were imported into a statistical processor (IBM-SPSS V25, IBM Corporate New York, NY, USA). Outliers were appropriately eliminated. Box plots and the Mann-Withney non-parametric U test were used to compare patients with no evidence of disease (NED) and relapsing (REL) patients. To evaluate the correlation between immunological parameters, a bivariate non-parametric Spearman correlation was performed.

Receiver operating characteristic (ROC) curves were used to analyze the predictive accuracy of the identified markers, considering both test specificity and sensitivity. ROC curves were generated by the IBM-SPSS processor, and cut-off values were generated by http://www.biosoft.hacettepe.edu.tr/easyROC/ online platform, applying the Youden method (40). The area under the curve (AUC), the asymptotic significance of the AUC and the 95% CI were used for evaluating predictive accuracy of the identified markers.

RFS was measured from the date of randomization until the date of relapse or death from any cause, and OS was measured from the date of randomization until death from any cause. For patients who were disease-free or alive at the time of data cutoff or for patients lost to follow-up, survival was censored on the last date of follow-up. The Kaplan–Meier method was used to estimate median survival, RFS, and OS distributions. The 95% confidence interval (CI) of these estimates was calculated [1.96 times the standard error (SE) in each direction]. Stratified log-rank test, at a two-sided α level of 0.05, was used to compare distributions of OS and RFS between treatment arms.

For the Principal Component Analysis (PCA), three continuous quantitative variables were used (CD56^dim^CD16^-^ NK, Treg and central memory γδ T cells) and 2 categorical variables: Arm (arm 1 and arm 2) and Outcome (REL and NED). PCA was applied only to patients having the complete set of readings of the selected parameters. Analyses were carried out by developing scripts, using functions and packages available in R language.

## Results

### Identification of baseline immune-related biomarkers by multi-parametric flow cytometry

In order to identify potential predictive biomarkers of response to our previously reported adjuvant treatment (vaccination in combination with IFN-α2b with or without dacarbazine) (15), peripheral blood mononuclear cells (PBMCs) of some patients (depending on the availability of samples) were *ex vivo* analyzed by multi-parametric flow cytometry. The analyses were performed before (pre), during (T92, i.e., at the fifth vaccination cycle), and after treatment (T4m and T6m, i.e., 4 and 6 months following randomization). Since the clinical outcome of patients receiving or not receiving dacarbazine was not significantly different, arm 1 and arm 2 patients were analyzed together (15).

A set of multi-color Ab panels (shown in **Supplementary Table S1**) was employed to assess the frequency, phenotype and functionality of several T and NK cell subsets. The gating strategies used for the analysis of the phenotype and function of the cell subpopulations examined are described in methods and **Supplementary Material** (**Supplementary Figures S1**, **S2**). A total of 368 cell subpopulations (listed in **Supplementary Tables S2**-**S4**) were evaluated by flow cytometry.

**Figure 1:**
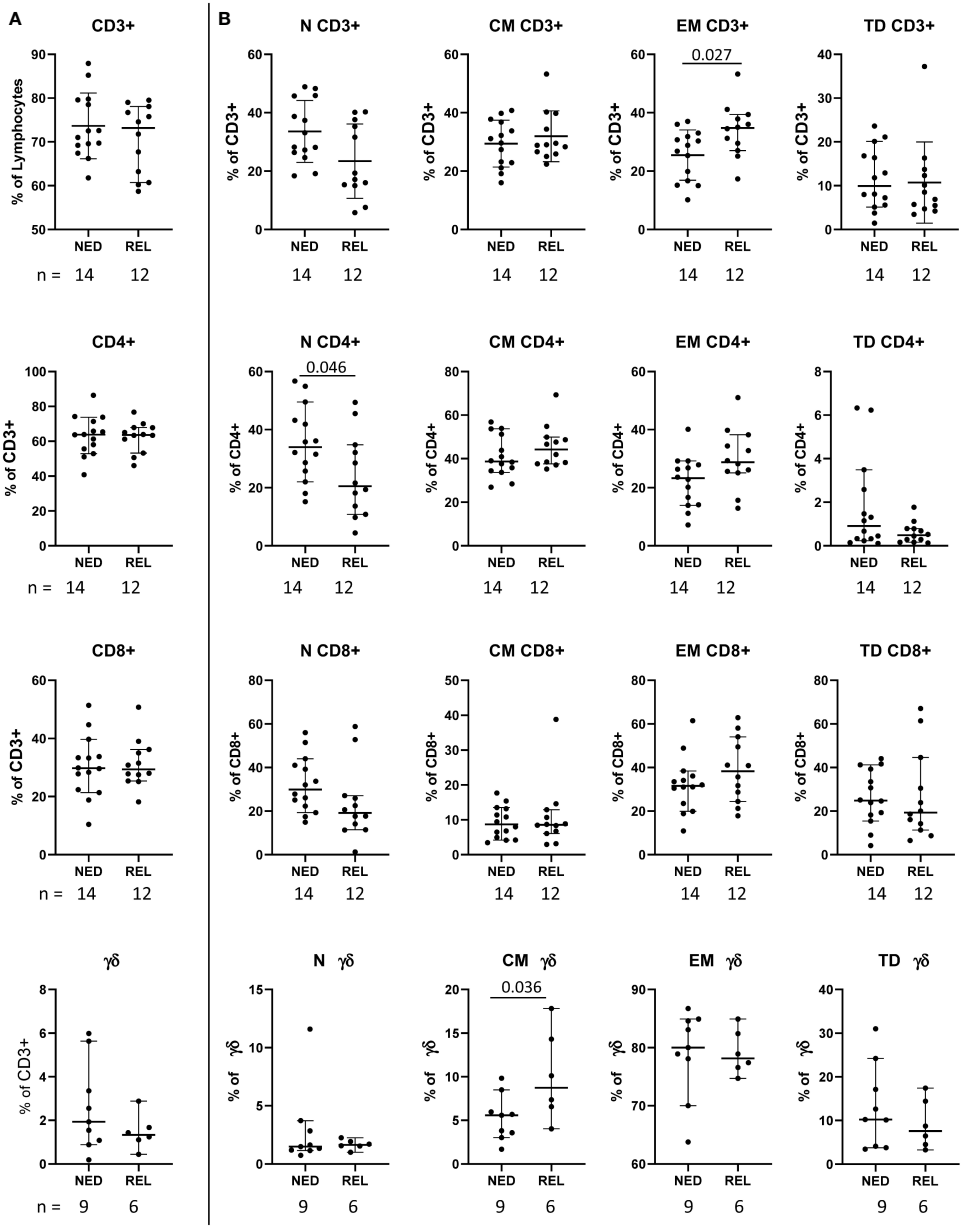
Pretreatment frequency of the main T cell subsets in relapsing (REL) and non-relapsing (NED) patients. Box plots (showing median, interquartile range, minimum and maximum) representing the pretreatment levels of the main T cell subsets in NED (no evidence of disease) and REL (relapsed) patients. The number of patients (n) is indicated below graphs. **(A)** Frequencies of CD3^+^ T cells (expressed as percentage of lymphocytes) and of CD4^+^, CD8^+^ and gd T cells (expressed as percentage of CD3^+^ cells). **(B)** Frequencies of naïve (N), central memory (CM), effector memory (EM) and terminally differentiated (TD) cells within the indicated T cell subset. P-values by Mann-Withney non-parametric U test.

Firstly, we compared the pretreatment frequencies of total CD3^+^ T cells and of the main T cell subsets (namely, CD4^+^, CD8^+^ and γδ T cells expressing the Vδ2 chain) in patients who recurred (REL) with respect to patients with no evidence of disease (NED) after treatment with surgery and combination therapy. As shown in **Figure 1A**, the frequency of total CD3^+^, CD8^+^, CD4^+^ and γδ T cells did not show any significant difference in patients with a different clinical outcome. For each T cell subset, the differentiation stages (gated as depicted in **Supplementary Figure S1C**) were dissected (**Figure 1B**). Interestingly, the baseline level of CD3^+^ T cells with an effector memory (EM) phenotype (CD45RA^−^CCR7^−^) was found significantly higher in REL than in NED patients (p ≤ 0.05 by independent non parametric Mann-Whitney U-test) (**Figure 1B**) accompanied by a trend of decrease of naïve (N) (CD45RA^+^CCR7^+^) CD3^+^ T cells (**Figure 1B**). Furthermore, a significant lower level of N CD4^+^ T cells was observed in REL than in NED patients (**Figure 1B**). An opposite tendency was observed in CD4^+^ T cells with EM phenotype, which showed a trend of increase in REL patients (**Figure 1B**). Although no significant differences were observed in any CD8^+^ subset, a trend of decrease of N CD8^+^ T cells and of increase of EM CD8^+^ T cells was observed in REL patients compared to patients who remained NED (**Figure 1B**).

The analysis of γδ T cell subpopulations showed that the level of central memory (CM) (CD45RA^-^CCR7^+^) γδ T cells was significantly higher in REL than in NED patients (**Figure 1B**).

**Figure 2:**
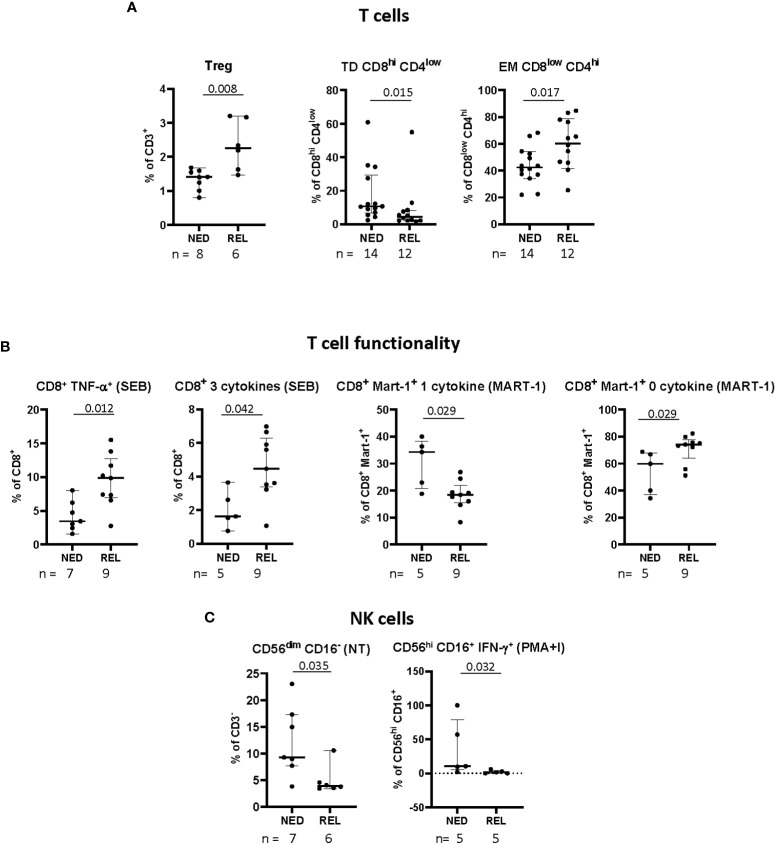
Pretreatment frequency and functionality of immune cell subsets in relapsing (REL) and non-relapsing (NED) patients. Box plots (showing median, interquartile range, minimum and maximum) represent 9 different immune cell subsets with a significantly different baseline frequency in NED and REL patients. The number of patients (n) is indicated below graphs. **(A)** Frequencies of regulatory T cells (Tregs, CD3^+^CD4^+^CD25^hi^CD127-Foxp3^+^), expressed as percentage of CD3^+^ T cells, of terminally differentiated (TD) CD8^hi^CD4^low^ (CD3^+^CD8^hi^CD4^low^CD45RA^+^CCR7-), and effector memory (EM) CD8^low^CD4^hi^ (CD3^+^CD8^low^CD4^hi^CD45RA-CCR7-), expressed as percentages of indicated subsets. **(B)** Polyfunctionality upon short-term in vitro expansion with staphylococcal enterotoxin B (SEB) or Melan-A peptide. Total CD8^+^ T cells producing TNF-α and 3 cytokines simultaneously in SEBstimulated samples (left panels). Melan-A-specific CD8^+^ T cells producing one and zero cytokines after Melan-A recognition (right panels). **(C)** Natural killer (NK) cell phenotype and functionality. The frequency of CD56^dim^CD16^-^ NK cell subset is expressed as percentage of CD3^-^ cells (left panel). Levels of CD56^hi^CD16^+^ NK cells producing IFN-γ in response to phorbol 12-myristate 13-acetate (PMA)/ionomycin (PMA^+^I), are shown as percentages of CD3^-^ cells (right panel). P-values by Mann-Withney non-parametric U test.

In addition to the principal T cell subsets, we analyzed also the frequency of CD3^+^ T cells expressing both CD4 and CD8 (double positive) and of CD3^+^ T cells not expressing neither CD4 nor CD8 (double negative) (gated as depicted in **Supplementary Figure S1B**). Among these subsets, a significant decrease of the frequency of CD8^hi^CD4^low^ double positive T cells with terminally differentiated phenotype (TD) (CD3^+^CD8^hi^CD4^low^ CD45RA^+^CCR7^-^) was observed in REL compared to NED patients (**Figure 2A**). On the contrary, the level of EM CD8^low^CD4^hi^ (CD3^+^CD8^low^CD4^hi^ CD45RA^-^CCR7^-^) was higher in REL than in NED patients (**Figure 2A**).

Treg cells were gated as depicted in **Supplementary Figure S1D** and identified as CD3^+^CD4^+^CD25^hi^CD127^-^Foxp3^+^. Remarkably, in patients relapsing after our vaccine-based combination therapy we observed significantly higher pre-treatment frequencies of Tregs (**Figure 2A**).

Then we analyzed the frequency of the baseline level of antigen-specific (Melan-A) CD8^+^ T cells (gated as depicted in **Supplementary Figure S2**), as well as the proportion of their naïve/memory subpopulations (**Supplementary Table S2**) without finding significant differences between REL and NED patients (data not shown).

Next, we investigated the functionality of total CD8^+^ T cells along with Melan-A^+^ CD8^+^ T cells, in terms of ability to express either none or one or more than one functional marker (namely, CD107a, TNF-α, IFN-γ, IL-2), in non-stimulated cells (NS), as well as in short-term *in vitro* stimulated cells (with either Melan-A antigen or SEB). The gating strategy is depicted in **Supplementary Figures S2A-C**. In total, 183 functional variables were analyzed (**Supplementary Table S3**). In **Figure 2B** only the subpopulations showing a statistically significant change of baseline frequency among REL and NED patients are shown. A higher baseline frequency of total CD8^+^ T cells producing TNF-α alone and producing 3 cytokines simultaneously in SEB-stimulated samples was observed in patients who later relapsed with respect to NED patients. Remarkably, when we analyzed CD8^+^ T-cell functionality inside Melan-A^+^ T lymphocytes we observed higher functionality in NED vs REL patients (**Figure 2B**, right panel).

Finally, different NK and NKT cell subsets and their functionality were analyzed as shown in **Supplementary Figure S2D**, up to 132 variables (**Supplementary Table S4**). The patients who remained NED after treatment showed a significantly higher proportion of CD56^dim^CD16^-^ NK cells at baseline, compared to REL patients. Moreover, in terms of functionality, the percentages of total NK and of CD56^hi^CD16^+^ NK cells producing IFN-γ in response to PMA and ionomycin were more elevated in NED than in REL patients (**Figure 2C**).

Summarizing the obtained results, among 368 analyzed variables we identified 12 different immune cell subsets whose pretreatment frequency is significantly associated to relapse after surgery and our adjuvant therapeutic strategy (**Figures 1**, **2**).

### Performance of baseline immune markers in predicting melanoma recurrence

**Figure 3:**
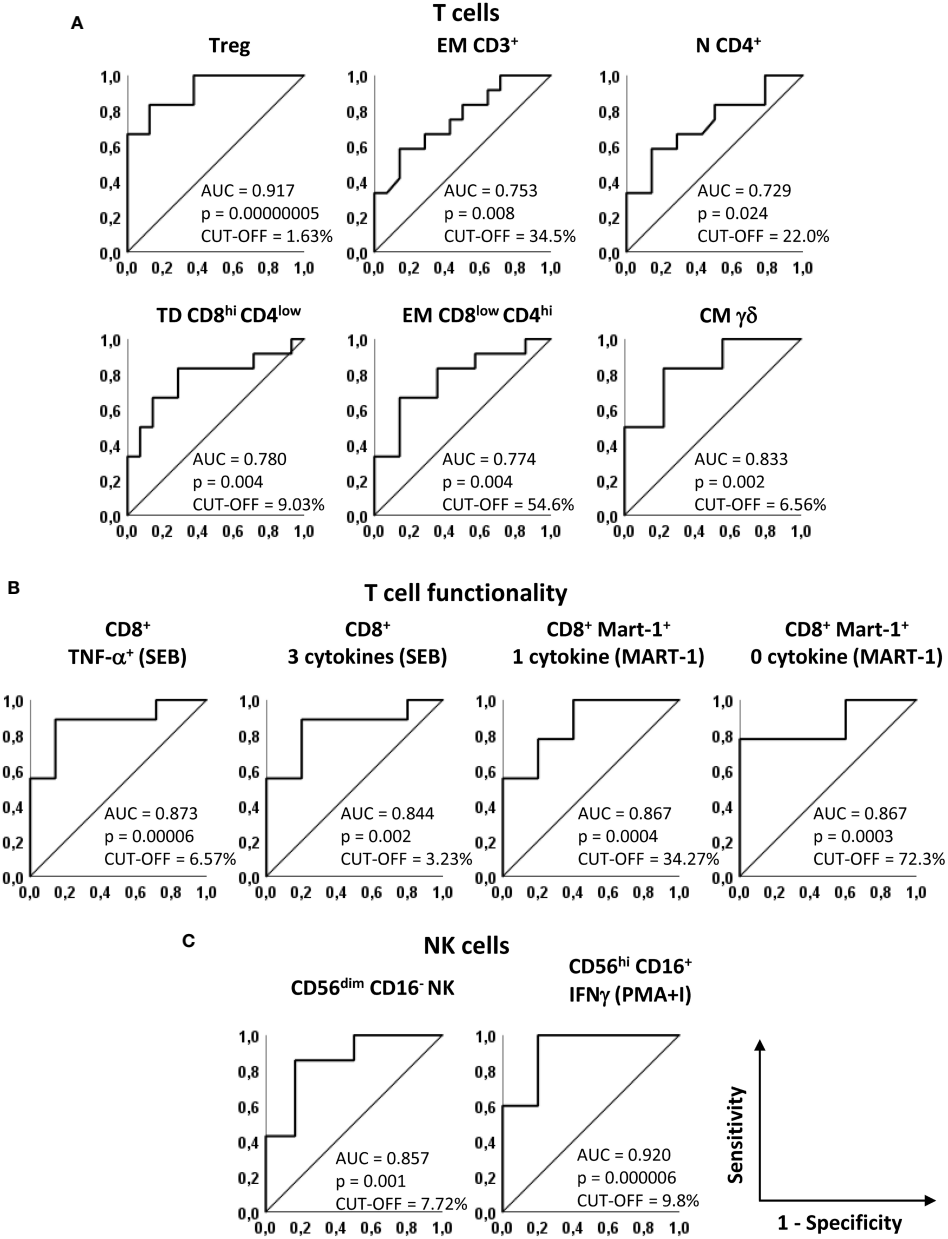
Predictive accuracy of the identified markers. Receiver operating characteristics (ROC) curves showing sensitivity and specificity of baseline markers that significantly discriminate NED and REL patients. **(A)** Treg, EM CD3^+^, N CD4^+^, TD CD8^hi^CD4^low^, EM CD8^low^CD4^hi^ and CM gd T cells, identified as in **Figures 1** and **2**. **(B)** SEB-stimulated CD8^+^ T cells producing TNF-α and 3 cytokines (left panels) and Melan-A-specific CD8^+^ T cells producing one and zero cytokines upon Melan-A peptide stimulation (right panels). **(C)** CD56^dim^CD16^-^ and PMA/ionomycin (PMA+I)-stimulated IFN-g^+^CD56^hi^CD16^+^ NK cells. The area under the curve (AUC), the asymptotic P-value and the cut-off are indicated for each curve.

We then used the receiver operating characteristic curve (ROC) method to evaluate the predictive ability of the 12 selected markers and to identify the cut-off values that best discriminate REL from NED patients. All 12 parameters showed a good performance in terms of area under the curve (AUC) (AUC > 0.7, p ≤ 0.05) (**Figure 3**). **Supplementary Tables S5** and **S6** report the curve coordinates the optimal cut-off values (Youden index) and the relative sensitivities and specificities for each marker.

Notably, 8 parameters showed AUC values higher than 0.8 in predicting recurrence in our patient cohort (**Figure 3**). In particular, among phenotypic markers, the AUC for baseline Treg frequency was 0.917 [Confidence interval (CI) 95% 0.767-1.066, p = 0.00000005] and the AUC for CM γδ T cells was 0.833 (CI 95% 0.619-1.048, p=0.002) (**Figure 3A**).

Among functional markers, the production of TNF-α and of 3 cytokines simultaneously in SEB-stimulated total CD8^+^ T cells showed AUCs of 0.873 (CI 95% 0.690-1.056, p=0.00006) and of 0.844 (CI 95% 0.627-1.062, p=0.002), respectively (**Figure 3B**). In addition, the analysis of cytokine production after Melan-A recognition, showed AUCs of 0.867 (CI 95% 0.665-1.068, p=0.0004) and 0.867 (CI 95% 0.669-1.065, p=0.0003) (**Figure 3B**).

For CD56^dim^CD16^-^ NK cells the AUC was 0.857 (CI 95% 0.643-1.072 p = 0.001) and for CD56^hi^ CD16^+^ IFN-γ^+^ NK cell subset the AUC was 0.920 (CI 95% 0.738-1.102, p=0.000006) (**Figure 3C**).

Despite the limitations due to the low number of patients, taken together, these results show that the analysis of baseline frequencies of all 12 immune cell subsets and in particular of the last 8 markers mentioned represents a sensitive approach for predicting which stage III/IV resected melanoma patient will be more likely to relapse after our combination adjuvant therapy.

### Kaplan-Meier survival analyses

**Figure 4:**
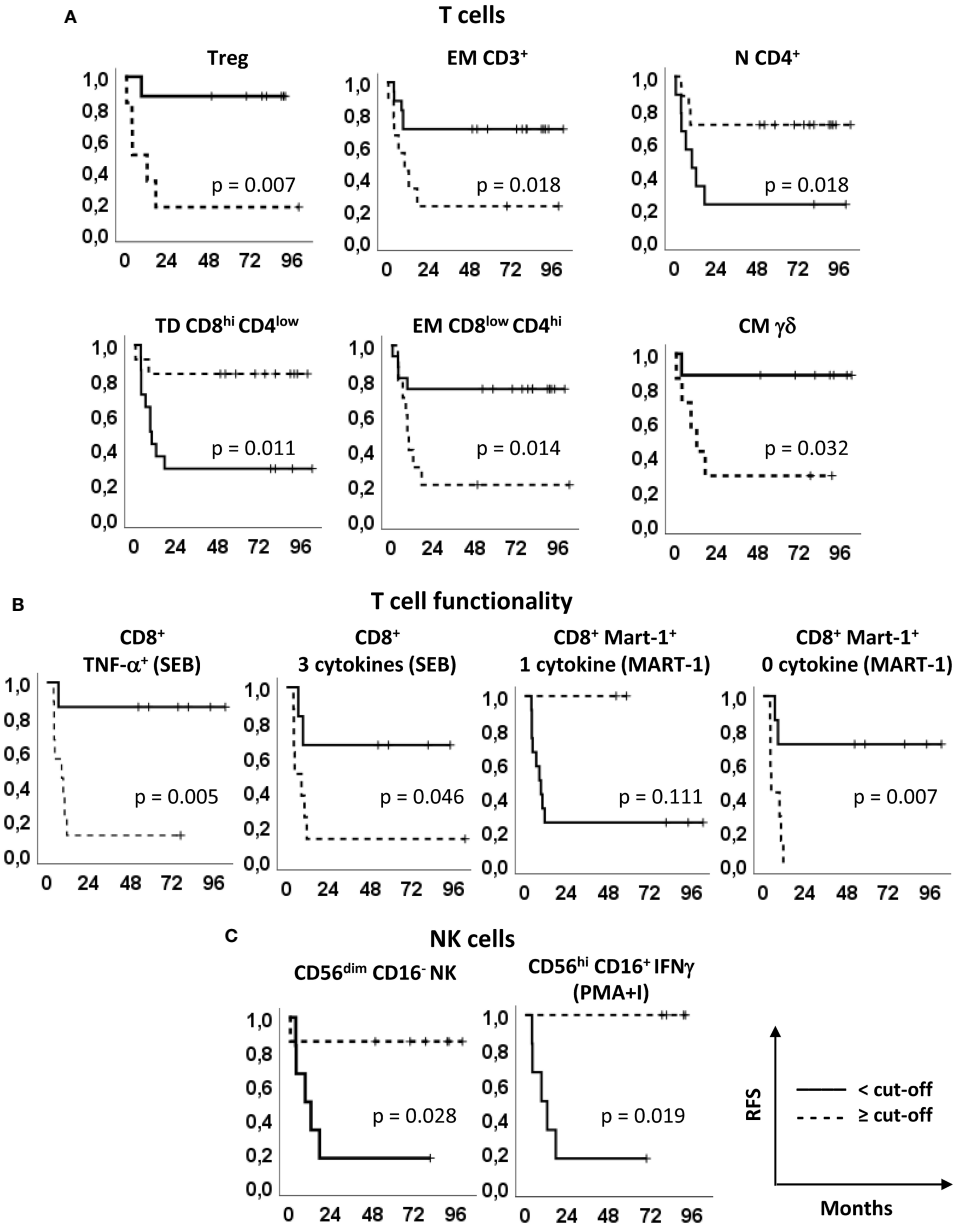
Relapse free survival analyses. Kaplan–Meier survival curves showing the relapse-free survival (RFS) of patients stratified according to the markers’ optimal cut-off identified by ROC analyses (see **Figure 2**, **Supplementary Tables S1** and **S2**). **(A)** Treg, EM CD3^+^, N CD4^+^, TD CD8^hi^CD4^low^, EM CD8^low^CD4^hi^ and CM γδ T cells, identified as in **Figures 1** and **2**. **(B)** SEB-stimulated total CD8^+^ T cells producing TNF-α and 3 cytokines (left panels) and Melan-A-specific CD8^+^ T cells producing one and zero cytokines upon Melan-A peptide stimulation (right panels). **(C)** CD56^dim^CD16^-^ and PMA/ionomycin (PMA+I)-stimulated IFN-γ^+^CD56^hi^CD16^+^ NK cells. Continue and dotted lines correspond to frequency < or ≥ cut-off, as indicated. P-values by log-rank test.

**Figure 5:**
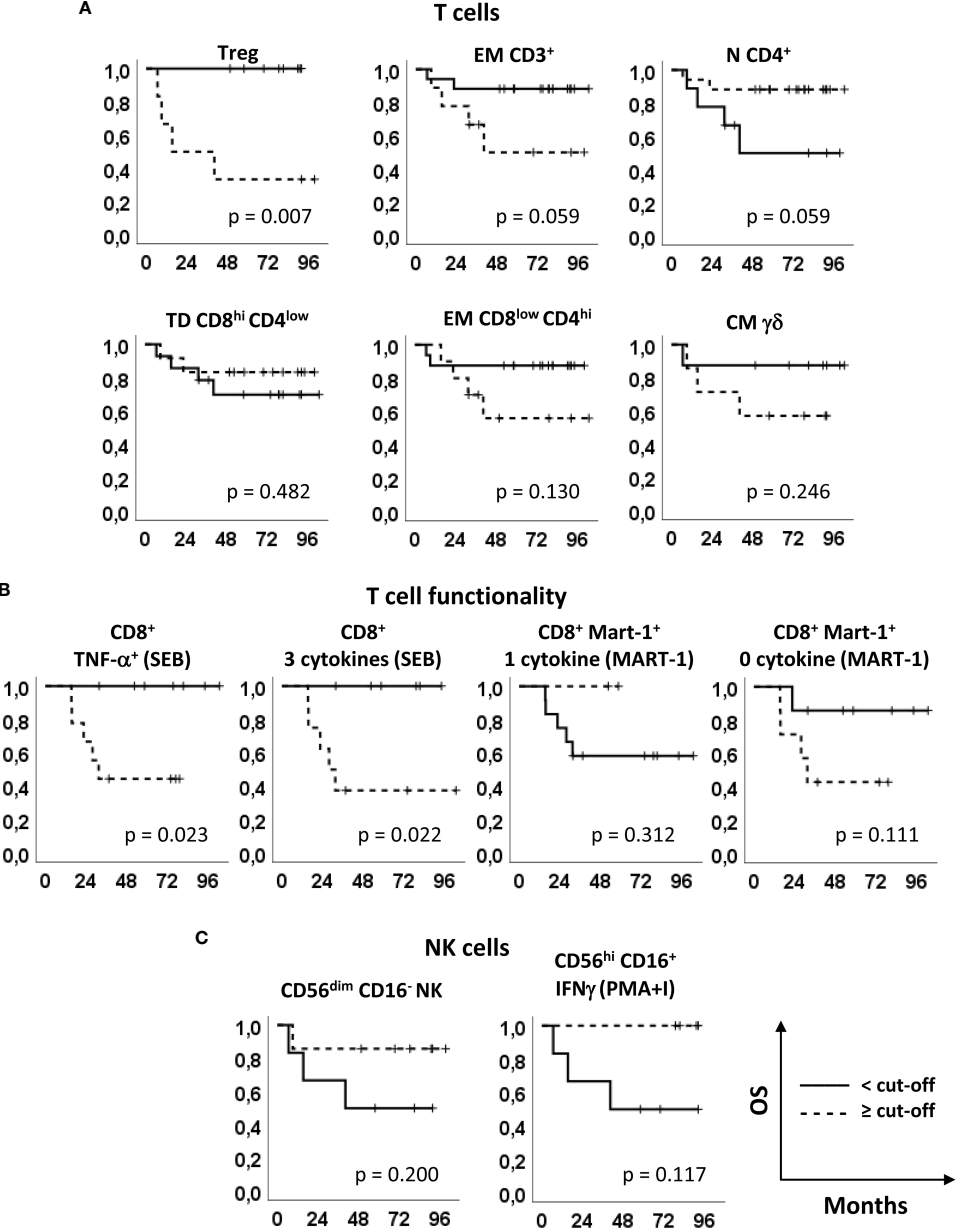
Overall survival analyses. Kaplan–Meier survival curves showing the overall survival (OS) of patients stratified according to the markers’ optimal cut-off identified by ROC analyses (see **Figure 2**, **Supplementary Tables S1** and **S2**). **(A)** Treg, EM CD3^+^, N CD4^+^, TD CD8^hi^CD4^low^, EM CD8^low^CD4^hi^ and CM γδ T cells, identified as in **Figures 1** and **2**. **(B)** SEB-stimulated total CD8^+^ T cells producing TNF-α and 3 cytokines (left panels) and Melan-A-specific CD8^+^ T cells producing one and zero cytokines upon Melan-A peptide stimulation (right panels). **(C)** CD56^dim^CD16^-^ and PMA/ionomycin (PMA+I)-stimulated IFN-γ^+^CD56^hi^CD16^+^ NK cells. Continue and dotted lines correspond to frequency < or ≥ cut-off, as indicated. P-values by log-rank test.

To better understand the relevance of these immune markers in the clinical outcome of the patients enrolled in our phase II clinical trial (15), the patients were stratified based on the optimal cut-off values indicated in **Supplementary Tables S5** and **S6** and Kaplan-Meier survival analyses were carried out (**Figures 4**, **5**). Noticeably, all but one parameters significantly predicted the RFS. The only non significant P-value was relative to the production of 1 cytokine by CD8^+^ Melan-A^+^ T cells, where the 2 patients with percentages higher than the cut-off showed a clear trend of long RFS but were lost early at follow-up (**Figure 4**). In our patient cohort, all recurrences occurred within the first 18 months; therefore, RFS did not vary from 18 months to the end of follow up (up to 8 years) (15). In particular, high levels of baseline circulating Tregs, EM CD3^+^ T cells, EM CD8^low^CD4^hi^ T cells, CM γδ T cells, TNF-α^+^ CD8^+^ T cells, CD8^+^ T cells producing 3 cytokines and CD8^+^ Melan-A^+^ T cells not producing cytokines were associated with a poor RFS. On the contrary, patients with high baseline levels of N CD4^+^ T cells, TD CD8^hi^CD4^low^ T cells, CD8^+^ Melan-A^+^ T cells producing 1 cytokine, CD56^dim^CD16^-^ NK cells and CD56^hi^ CD16^+^ NK cells IFN-γ^+^ showed a good prognosis. Noticeably, the baseline levels of Tregs and of SEB-stimulated CD8^+^ T cells producing TNF-α and 3 cytokines significantly (p ≤ 0.05) predicted the OS (**Figure 5**).

These results suggest that the baseline levels of 11 immune biomarkers identified by multi-parametric flow cytometry correlate with the RFS and 3 of them correlate also with the OS of resected melanoma patients undergoing treatment with peptide-based vaccination and IFN-α (with or without dacarbazine).

### Predictive power of biomarker combination

We then focused our attention on 3 subsets, namely Tregs, γδ CM T cells and CD56^dim^CD16^-^ NK cells, showing an AUC>8 and that can be analyzed through surface staining assay, which is a simple testing method within the reach of non-specialized analysis laboratories.

A multivariate analysis was carried out using PCA to evaluate whether these 3 parameters might effectively group the patients according to the clinical outcome.

**Figure 6:**
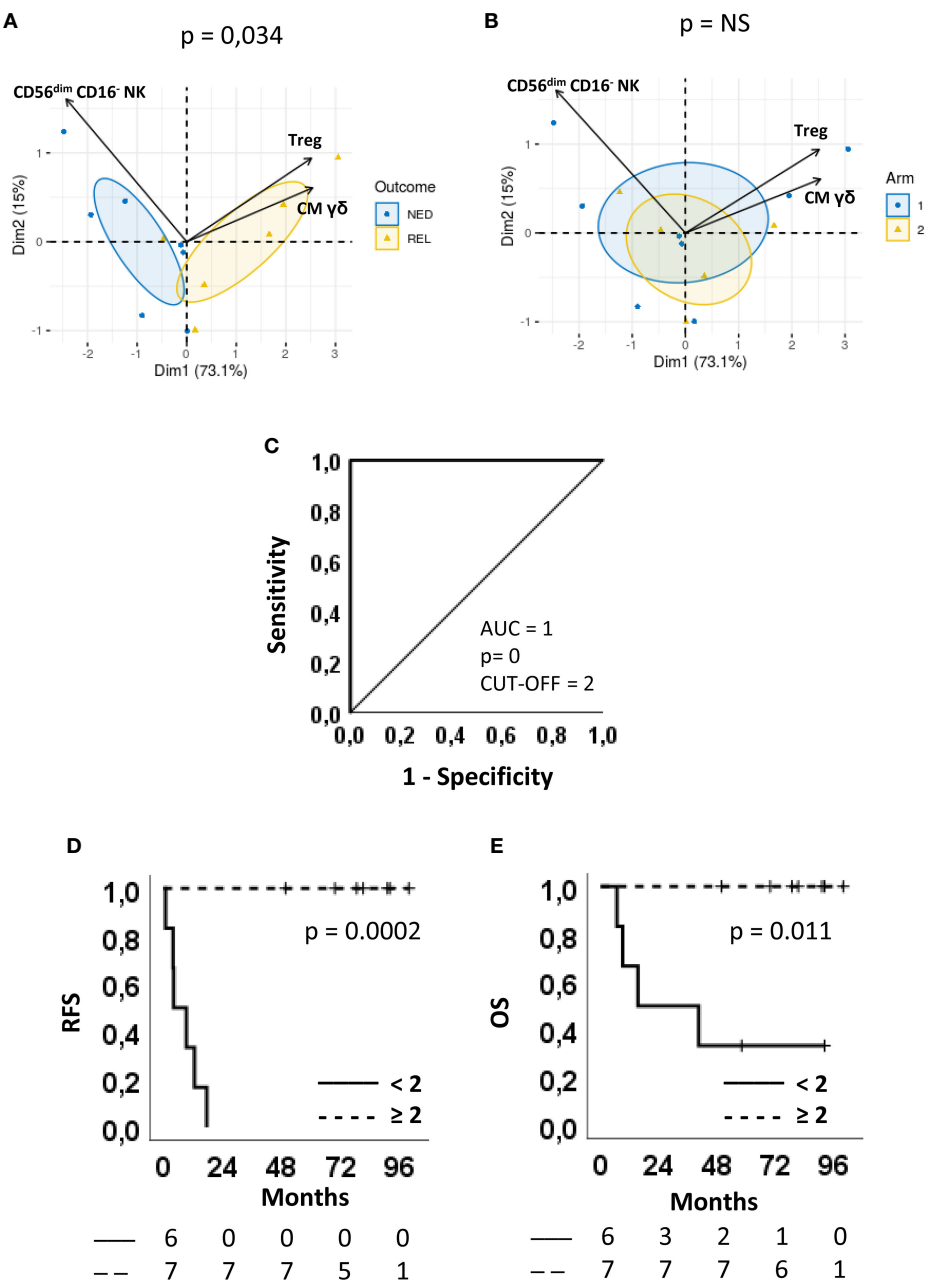
Predictive accuracy of the combination score and survival curves. **(A, B)** Multivariate analysis by principal component analysis (PCA) of the three parameters selected for the combination score, regulatory T cells (Treg), CD56^dim^CD16^-^ NK cells and CM γδ T cells. Biplots displaying both patients (points) and parameters (vectors). Confidence ellipses are provided (CI = 95%) for patients after grouping them according to either **(A)** Clinical outcome (blue circle=NED, yellow triangle=REL) or **(B)** Arm (blue circle=arm 1, yellow triangle=arm 2). In the component space, separation between individuals grouped by Arm (p-value=0.78) or Outcome (p-value=0.034) was evaluated using Wilks test. **(C)** ROC curves showing sensitivity and specificity of the combination score. The area under the curve (AUC), the asymptotic P-value, and the optimal cut-off are indicated. **(D, E)** Kaplan-Meier plots showing **(D)** the relapse-free survival (RFS) and **(E)** the overall survival (OS) of patients stratified according to the cut-off. Continue and dotted lines correspond to frequency < or ≥ cut-off, as indicated. The number of patients at risk is indicated below D and E plots according to the patient stratification. P-values by log-rank test.

PCA generated a two-component solution accounting for 88.1% of explained global variance; the original variables were most represented by Dim1, which explained 73.1% of variance. On the one hand, a complete discrimination of the two patient groups (**Figure 6A**) was revealed along Dim1. On the other hand, no significant grouping of individuals occurred in the component space according to the treatment arm, thus confirming the absence of any bias on the clinical outcome (**Figure 6B**).

On Dim1, the levels of CM γδ T cells and Treg had positive loadings (0.587 and 0.581, respectively) whereas the levels of CD56^dim^CD16^-^ NK cells had a negative loading (-0.562). Patients with positive coordinate (REL) are characterized by higher frequencies of Treg and CM γδ T cells, whereas those with negative coordinate (NED) by higher frequencies of CD56^dim^CD16^-^ NK cells. This implies that the discrimination between NED e REL outcomes rely on the balance between CM γδ T cell and Treg *vs* CD56^dim^CD16^-^ NK cell proportions.

Interestingly, the levels of Tregs did not change upon treatment (**Supplementary Figure S3A**). The lower levels of CD56^dim^CD16^-^ NK cells observed at baseline in REL vs. NED patients showed a tendency (albeit not significant) to be maintained at T92 (**Supplementary Figure S3B**). Nevertheless, 4 and 6 months following patient randomization there was no difference between REL and NED patients. Similarly to Treg cells, CM γδ T lymphocytes were also significantly more abundant in REL than in NED subjects before treatment and this difference was maintained during the course of treatment and beyond, showing that these immune cell subsets are not influenced by treatment (**Supplementary Figure S3C**).

We then assessed whether the combination of the three immune biomarkers could increase the ability of predicting the clinical outcome of our patient cohort. As shown in **Table 1**, a scoring system was applied to the frequency of each immune cell subset. For Treg and CM γδ T cells a score = 1 was attributed to patients showing percentages of cells < of their respective cut-off and a score = 0 was given to patients with the opposite immunophenotype. On the contrary, a score = 1 was given to patients with a frequency of CD56^dim^CD16^-^ NK cells ≥6.14% and a score = 0 to patients with a frequency <6.14%. Considering only patients that were analyzed for all the three variables (NED n=7; REL n=6), a combination score for each of them was obtained by adding the three scores. As shown in the last column of **Table 1**, all REL patients had a score equal to either 0 or 1, i.e. were identified by 2 out of the 3 identified immune biomarkers. Conversely, all patients who remained NED following surgery and treatment had a combined score equal to 2 or 3. This analysis show that the combination of the three markers has a higher predictive value than each immune biomarker by itself in our setting. Of note, no correlation was found with the treatment arm.

Table 1Combination score.

**Table:** 

			Treg		CD56^dim^ CD16^-^ NK		CM Vδ2 CD3^+^		
		cut off	**1,63%**		**7,72%**		**6,56%**		
		< cut off	1		0		1		
		≥ cut off	0		1		0

**Table:** 

Patient random_no	Arm	Clinical Outcome	%	Score	%	Score	%	Score	Combination Score
002	2	NED	1,68	0	14,97	1	3,58	1	2
005	1	NED	0,8	1	17,3	1	5,57	1	3
007	1	NED	1,59	1	9,29	1	8,49	0	2
010	1	NED	1,41	1	23,02	1	1,69	1	3
017	2	NED	1,54	1	3,81	0	5,95	1	2
026	1	NED	1,24	1	7,72	1	3,8	1	3
028	1	NED	1,4	1	8,96	1	9,82	0	2
011	2	REL	2,19	0	4,06	0	14,3	0	0
013	1	REL	2,33	0	4,55	0	4,03	1	1
015	1	REL	1,47	1	3,51	0	7,35	0	1
022	1	REL	3,2	0	3,41	0	17,8	0	0
031	2	REL	3,16	0	3,76	0	10,1	0	0
034	2	REL	1,63	0	10,58	1	6,56	0	1

To further confirm this observation, a ROC curve was produced using the combination score. Remarkably, the resulting AUC was 1 (CI 95% 1.000-1.000, p = 0) and the sensitivity and specificity of the test were both 100%, by using a cut-off = 2 (**Figure 6C**).

The combination score was also able to significantly separate Kaplan-Meier RFS (P=0.0002) (**Figure 6D**) and OS (P=0.011) curves (**Figure 6E**). In fact, 100% of patients with a combination score ≥ 2 survived with no recurrences or death, while all patients with a combination score < 2 recurred within 18 months (**Figure 6D**) and 4 out of 6 patients died within 40 months from randomization (**Figure 6E**).

These results show that the three cell subsets Treg, CD56^dim^CD16^-^ NK and γδ T cells, alone or more precisely when combined, may represent circulating biomarkers able to predict clinical response in melanoma patients treated with vaccine-based therapy.

## Discussion

Personalized medicine is the last frontier of medical science. To move towards this, it is crucial to identify biomarkers predicting the patient response, thus targeting the right therapy to the most suitable patient.

Blood biomarkers represent particularly useful candidates, since venous sampling is a much less invasive practice than surgery or biopsy.

In the present study, the pre-therapy immunological status in the peripheral blood of patients with resected melanoma, undergoing an experimental combined immunotherapy, was investigated to underscore the association between the frequency of different immune cell subsets/functionality at baseline and the clinical outcome, with the final intent to determine a “peripheral immunoscore” with a prognostic significance (36).

The immunophenotype of T lymphocytes demonstrated that patients showing tumor relapse after surgery and our combination immunotherapy were characterized by baseline higher levels of Treg cells, EM CD3^+^ T cells, EM CD8^low^CD4^hi^ double positive T cells and CM γδ T cells and lower levels of N CD4^+^ T cells and TD CD8^hi^CD4^low^ double positive T cells, with respect to patients with a favorable outcome.

Tregs suppress the immune response and as such may suppress the response to cancer vaccines and to immunoregulatory therapies, such as IFN-α. Indeed, accumulation of Treg has been frequently reported both in the tumor microenvironment and in the peripheral blood of advanced melanoma patients, representing a dominant mechanism of tumor immune evasion and a major obstacle for cancer immunotherapy (41). Less studied is the frequency of this cell subset after complete tumor resection and its impact on the efficacy of immunotherapy. In this regard, Ascierto and colleagues demonstrated significantly higher baseline Treg levels for stage III vs. stage IV disease and early recurrence vs. no recurrence in melanoma patients treated in adjuvant with high-dose IFN-α2b (42), indicating that this subset plays a key role in the response to IFN-α, whose mechanism of action relies indeed more on an indirect immunoregulatory mechanism (27) (which can be hampered by Tregs) rather than on a direct anti-tumor effect.

The memory phenotype of T cells is a consequence of the history of antigen encounter, acute vs. chronic exposure to antigen and co-stimulation. Previous studies showed a correlation between the memory phenotype of blood T cells and the therapeutic efficacy of checkpoint inhibitor-based immunotherapy (43). In the present study, patients that relapsed after surgery and adjuvant treatment showed increased EM T cells and, conversely, less N T cells compared to NED patients. Typically, cancer patients exhibit a relative decrease in N and CM T cells and an increase of EM and TD T cells (44). In accordance to our findings, several studies have demonstrated that CM T cells have a superior persistence and antitumor immunity compared with EM T cells and effector T cells (45).

In addition to CD3^+^ EM T cells, we found that also CD8^low^CD4^hi^ double positive T cells with an EM phenotype were higher in REL than in NED patients. The presence of double positive CD4^+^ CD8^+^ T cells was demonstrated in metastatic lesions and lymph nodes of melanoma patients (46), as well as in other cancer types, but was more rarely described in the peripheral blood. Very little is known about their antigen specificity and their functions, besides that they exhibit cytolytic activity along with T helper cytokine expression. To the best of our knowledge, this is the first report showing a predictive ability of blood double positive T cells in the clinical outcome of patients with melanoma.

Accumulating evidence suggests an important role of γδ T cells in the anti-tumor response. These cells can kill tumor cells in an MHC-unrestricted manner, and possess potential regulatory capability and antigen-presenting capacity. Low frequencies of circulating Vδ2^+^ γδ T cells and high proportions of Vδ1^+^ γδ T cells were reported in melanoma patients (47) and a high baseline frequency of Vδ2^+^ γδ T cells was shown to correlate with longer OS in ipilimumab-treated patients, but only minor differences in the differentiation phenotype were observed (48). We identified CM Vδ2^+^ γδ T cells as an early indicator of worst clinical outcome in resected melanoma patients undergoing our combination immunotherapy. γδ T cells with a TD phenotype have increased cytotoxic potential and limited cytokine production. On the contrary, cells with N or CM phenotypes lack immediate effector function and cytotoxic activity. A high proportion of circulating γδ T cells in a naïve stage was associated with an early relapse of melanoma patients, suggesting that the lack of differentiated γδ T cells is predictive of a poorer outcome in melanoma (49). It remains to be determined why, unlike total CD3^+^ T cells, in which the EM phenotype prevails in relapsing patients, in the case of γδ T cells the CM phenotype prevails.

Functional assays were also performed to assess the polyfunctionality of vaccine-specific and total CD8^+^ T cells. Prior to treatment, total CD8^+^ T cells of patients who later relapsed were more polyfunctional (producing simultaneously 3 cytokines) and produced more TNF-α in response to SEB stimulation than cells from NED patients. Nevertheless, Melan-A-specific T cells were more anergic in response to Melan-A in REL than in NED patients, suggesting that the functional profile of T cells in response to the cognate antigen can represent an important parameter to be monitored before enrolling a patient in a vaccination trial.

Since their central role in anti-tumor immunity, we measured also the impact of the frequency of different NK cell subsets and of their ability to produce IFN-γ after stimulation with PMA and ionomycin. The frequency of CD56^dim^CD16^-^ NK, which among NK subsets display an intermediate maturation level (in terms of proliferating and cytotoxic activity), was shown to be higher in non-relapsing than in relapsing patients, confirming previous data showing the association of this NK subset with better clinical outcome in melanoma patients treated with vaccination and high-dose IFN-α2b (50). This NK subset lacks the ability to perform antibody-dependent cell-mediated cytotoxicity due to lack of CD16 expression and is believed to exert its anti-tumor activity through secreted factors such as cytokines, chemokines, and growth factors, which may in turn induce an inflammatory response sustaining T cell-mediated immunity, and possibly the response to the vaccine. On the other hand, IFN-α is able to increase the cytotoxic functions of NK cells, further stimulating their anti-tumor activity (51).

Moreover, the baseline levels of CD56^hi^CD16^+^ NK, expressing IFN-γ following PMA and ionomycin, were higher in patients who remained NED than in recurring patients. Notably, dacarbazine was shown to upregulate the expression of NKG2D ligands on tumor cells, thus promoting NK-cell cytotoxicity and IFN-γ secretion (52), which might have further enhanced the role exerted by NK cells in arm 2 patients.

Taken individually, 11 identified circulating immune markers showed a good predictive ability of the predisposition to relapse and 3 of them (i.e. Treg, 3 cytokine^+^ CD8^+^ T cells and TNF-α^+^ CD8^+^ T cells) correlated also with the OS. Noteworthy, the best performance was obtained with the combination of 3 easily analyzable markers, which showed an AUC of 1 and an impressive ability to distinguish low and high-risk patients. The predictive score stratified patients into high- (score ≥2) and low- (score <2) risk groups, showing significant differences in both RFS and OS.

The prognostic/predictive role of peripheral blood immune-related biomarkers has been recently explored in different cancer immunotherapy settings (53, 54). Various circulating lymphoid and myeloid immune cell subsets have been identified, as potential baseline biomarkers in patients treated with ICI (55), mostly in patients with melanoma (43) and lung cancer (56, 57).

As an example, a combination of four NK and T cell subsets (Granzyme B^+^ NK cells, CD4^+^ effector T cells (CD4^+^CD45RA^+^CD27^−^), CD45RA^+^CCR7^+^ TNFα^+^ CD4^+^ T cells and HLADR^−^CD38^−^ CD4^+^ T cells were determined as sensitive and specific biomarkers of response to anti-CTLA-4 (AUC of 0.729). The same authors showed that NK cell subsets (but not memory T cell subsets) correlated with clinical response to anti-PD-1 therapy (43). In non-small cell lung cancer patients, high levels of circulating CD4^+^ T cells, NK cells and Tregs were shown to predict the response to anti-PD-1 (56, 57). A baseline immune signature has been found in patients with different metastatic solid tumors benefiting from ICI. De Lima and collaborators elaborated a numerical index by dividing the relative counts of CD8^+^PD1^+^, CD8^+^ EM cells and DCs by mMDSCs and classical monocytes, which showed a good predictive accuracy (AUC of 0.845) (58). In patients with different solid tumors a peripheral blood immune cell-based signature showed an excellent accuracy in predicting 0.5-, 1-, and 2-year OS upon ICI treatment (AUC = 0.657, 0.701, and 0.746, respectively) (59).

The research of markers associated with the response to a certain therapy may be exploited in three different ways: i) as a mechanism‐based approach to tailor treatment to the patients with more chances to benefit from it (e.g. to select patients with less immunosuppression for immunotherapy trials); ii) as a method to develop mechanism-based novel combination therapies and ii) as an additional tool for patient management (e.g. intensification of the instrumental diagnostic program in patients with the greatest risk of recurrence).

Risk stratification is particularly important in the setting of the currently used adjuvant treatment of melanoma, for both toxicities and costs related to immune checkpoint inhibitors and targeted therapies. In these contexts, high tumor mutational burden (TMB), CD8^+^ T-cell tumor infiltrate and IFN-γ expression were found to correlate with favorable clinical outcomes with nivolumab and ipilimumab (60). Conversely, low TMB was found to correlate with longer RFS upon treatment with dabrafenib plus trametinib, a discrepancy potentially related to the fact that in targeted therapies the genetic heterogeneity is potentially related to increased tumor escape mechanisms (60). In our trial the patients were enrolled after surgery, therefore we could not analyze neither the tumor nor the TME characteristics. Nevertheless, similarly to target therapies, it is possible to speculate that tumor associated antigens-based vaccines may be more beneficial for patients with low tumor mutational burden, because of reduced risk of tumor escape by antigen loss.

The present study exhibits obvious limitations. In spite of the statistical significance of some of the data reported herein, the patient cohort was small and the findings need to be validated in independent patient cohorts with a high number of patients. Moreover, it remains to be clarified whether at least some of the identified markers are predictive of clinical response also to other adjuvant therapies and whether they can represent prognostic markers in patients not undergoing treatment. In spite of these limitations, this study provides the rationale for the evaluation of several immune cell subsets to help determine correlates of clinical outcome in resected melanoma patients with high-risk of recurrence and suggests that a combination of indicators could be more accurate than single ones, opening new perspectives for research challenges aimed towards a personalized cancer immunotherapy.

## Conclusions

We identified and evaluated different circulating immune markers and a combination score potentially capable of predicting recurrence and death of completely resected melanoma patients undergoing an experimental adjuvant combined immunotherapy. The findings suggest that single indicators and the combination score could be promising non-invasive biomarkers in melanoma patients with high risk of recurrence.

## Data availability statement

The raw data supporting the conclusions of this article will be made available by the authors, without undue reservation.

## Ethics statement

The studies involving human participants were reviewed and approved by IRCCS Regina Elena National Cancer Institute, Ethics Committee. The patients/participants provided their written informed consent to participate in this study.

## Author contributions

FM, FU, IM and EP designed the study. IM, FU, FM, BP and PN developed the methodologies. VF provided and managed patients. IM, FU, BP, LC, CB, AG and FI acquired data. IR, FU and IM performed statistical analyses and data interpretation. FM wrote the manuscript. FM, IM, FU, LC, FB, BP, PN, VF and EP reviewed the manuscript. All authors contributed to the article and approved the submitted version.
