# Supplementary material for: Blood immune cells as potential biomarkers predicting relapse-free survival of stage III/IV resected melanoma patients treated with peptide-based vaccination and interferon-alpha
Source: Front Oncol. 2023 May 18;13:1145667. doi: 10.3389/fonc.2023.1145667 (PMC10233106; doi:10.3389/fonc.2023.1145667)
Supplement: Supplementary file 1 [file DataSheet_1.pdf]

Supplementary Table S1: Flow Cytometry Panels

Staining panel	Antigen/Ligand	Clone	Fluorochrome	Species	Manufacturer	Nationality
Major and TCR γδ T lymphocyte subpopulation memory-naïve phenotype	CD45RA	HI100	FITC	mouse	Biolegend	California
	TCR Vd2	B6	PE	mouse	BD Pharmingen	California
	CCR7 (CD197)	G043H7	PerCP Cy5.5	mouse	BD Pharmingen	California
	CD8	SK1	PE Cy7	mouse	BD Pharmingen	California
	CD4	L200	APC	mouse	BD Pharmingen	California
	CD3	SK7	APC H7	mouse	BD Pharmingen	California
Treg frequency	CD4	RPA-T4	FITC	mouse	BD Pharmingen	California
	Foxp3	PCH101	PE	rat	e-Bioscience	Massachusetts
	CD3	SP34-2	PerCP Cy5.5	mouse	BD Pharmingen	California
	CD25	M-A251	PE Cy7	mouse	BD Pharmingen	California
	CD127	HIL-7R-M21	Alexa Fluor 647	mouse	BD Pharmingen	California
	Dead cells	NA	nIR	NA	Molecular Probes	Oregon
Total CD8+ cell and MART-1+ CD8+ cell functionality	CD107a	H4A3	FITC	mouse	BD Pharmingen	California
	MART-1 Tetramer	NA	PE	NA	Beckman Coulter	California
	TNF-a	Mab11	PerCP Cy5.5	mouse	e-Bioscience	Massachussetts
	IFN-g	B27	PE Cy7	mouse	BD Pharmingen	California
	IL-2	MQ1-17H12	APC	rat	BD Pharmingen	California
	CD8	SK1	APC H7	mouse	BD Pharmingen	California
NK subpopulation and NKT cell phenotype and functionality	CD107a	H4A3	FITC	mouse	BD Pharmingen	California
	CD56	B159	PE	mouse	BD Pharmingen	California
	CD3	SP34-2	PerCP Cy5.5	mouse	BD Pharmingen	California
	IFN-g	B27	PE Cy7	mouse	BD Pharmingen	California
	CD16	LNK16	Alexa Fluor 647	mouse	Serotec	California
	Dead cells	NA	nIR	NA	Molecular Probes	Oregon

Supplementary Table S2

Major and TCR γδ T lymphocyte subpopulation memory-naive phenotype Panel (46 variables)

parent population	Variable
Singlets	Lymphocytes
Lymphocytes	CD3+
	TCR Vδ2+
CD3+	CD4+
	CD8+
	CD8hi CD4lo
	CD8lo CD4hi
	CD4CD8DN
CD3+	CD8+ MART+
	CD8+ MARThi
CD3+	CD45RA+ CCR7+ (N)
	CD45RA- CCR7+ (CM)
	CD45RA- CCR7- (EM)
	CD45RA+ CCR7- (EM)
TCR Vδ2+	CD45RA+ CCR7+ (N)
	CD45RA- CCR7+ (CM)
	CD45RA- CCR7- (EM)
	CD45RA+ CCR7- (EM)
CD4+	CD45RA+ CCR7+ (N)
	CD45RA- CCR7+ (CM)
	CD45RA- CCR7- (EM)
	CD45RA+ CCR7- (EM)
CD8+	CD45RA+ CCR7+ (N)
	CD45RA- CCR7+ (CM)
	CD45RA- CCR7- (EM)
	CD45RA+ CCR7- (EM)
CD8hi CD4lo	CD45RA+ CCR7+ (N)
	CD45RA- CCR7+ (CM)
	CD45RA- CCR7- (EM)
	CD45RA+ CCR7- (EM)
CD8lo CD4hi	CD45RA+ CCR7+ (N)
	CD45RA- CCR7+ (CM)
	CD45RA- CCR7- (EM)
	CD45RA+ CCR7- (EM)
CD4CD8DN	CD45RA+ CCR7+ (N)
	CD45RA- CCR7+ (CM)
	CD45RA- CCR7- (EM)
	CD45RA+ CCR7- (EM)
CD8MART+	CD45RA+ CCR7+ (N)
	CD45RA- CCR7+ (CM)
	CD45RA- CCR7- (EM)
	CD45RA+ CCR7- (EM)
CD8MARThi	CD45RA+ CCR7+ (N)
	CD45RA- CCR7+ (CM)
	CD45RA- CCR7- (EM)
	CD45RA+ CCR7- (EM)

Treg frequency (4 variables)

parent population	Variable
Lymphocytes	FoxP3+ CD127- (Treg)
CD3+	FoxP3+ CD127- (Treg)
CD4+	FoxP3+ CD127- (Treg)
CD4+CD25hi	FoxP3+ CD127- (Treg)

Tetramer staining (3 variables)

parent population	Variable
CD8+	ex vivo MART-1 +
	in vitro MART-1 +
	in vitro NY-ESO-1 +

Supplementary Table S3

Total CD8+ and MART-1+CD8+ cell functionality (183 variables)

culture	parent population	Variable	culture	parent population	Variable	culture	parent population	Variable
NS	Singlets	Lymphocytes	MART-1	Singlets	Lymphocytes	SEB	Singlets	Lymphocytes
	Lymphocytes	CD8+MART-1+		Lymphocytes	CD8+MART-1+		Lymphocytes	CD8+MART-1+
		CD8+			CD8+			CD8+
	CD8+MART-1+	CD107a+		CD8+MART-1+	CD107a+		CD8+MART-1+	CD107a+
		IFNg+			IFNg+			IFNg+
		IL2+			IL2+			IL2+
		TNFa+			TNFa+			TNFa+
		CD107a-			CD107a-			CD107a-
		IFNg-			IFNg-			IFNg-
		IL2-			IL2-			IL2-
		TNFa-			TNFa-			TNFa-
		CD107a+IFNg+IL2+TNFa+			CD107a+IFNg+IL2+TNFa+			CD107a+IFNg+IL2+TNFa+
		CD107a+IFNg+IL2+TNFa-			CD107a+IFNg+IL2+TNFa-			CD107a+IFNg+IL2+TNFa-
		CD107a+IFNg+IL2-TNFa+			CD107a+IFNg+IL2-TNFa+			CD107a+IFNg+IL2-TNFa+
		CD107a+IFNg+IL2-TNFa-			CD107a+IFNg+IL2-TNFa-			CD107a+IFNg+IL2-TNFa-
		CD107a+IFNg-IL2+TNFa+			CD107a+IFNg-IL2+TNFa+			CD107a+IFNg-IL2+TNFa+
		CD107a+IFNg-IL2+TNFa-			CD107a+IFNg-IL2+TNFa-			CD107a+IFNg-IL2+TNFa-
		CD107a+IFNg-IL2-TNFa+			CD107a+IFNg-IL2-TNFa+			CD107a+IFNg-IL2-TNFa+
		CD107a+IFNg-IL2-TNFa-			CD107a+IFNg-IL2-TNFa-			CD107a+IFNg-IL2-TNFa-
		CD107a-IFNg+IL2+TNFa+			CD107a-IFNg+IL2+TNFa+			CD107a-IFNg+IL2+TNFa+
		CD107a-IFNg+IL2+TNFa-			CD107a-IFNg+IL2+TNFa-			CD107a-IFNg+IL2+TNFa-
		CD107a-IFNg+IL2-TNFa+			CD107a-IFNg+IL2-TNFa+			CD107a-IFNg+IL2-TNFa+
		CD107a-IFNg+IL2-TNFa-			CD107a-IFNg+IL2-TNFa-			CD107a-IFNg+IL2-TNFa-
		CD107a-IFNg-IL2+TNFa+			CD107a-IFNg-IL2+TNFa+			CD107a-IFNg-IL2+TNFa+
		CD107a-IFNg-IL2+TNFa-			CD107a-IFNg-IL2+TNFa-			CD107a-IFNg-IL2+TNFa-
		CD107a-IFNg-IL2-TNFa+			CD107a-IFNg-IL2-TNFa+			CD107a-IFNg-IL2-TNFa+
		CD107a-IFNg-IL2-TNFa-			CD107a-IFNg-IL2-TNFa-			CD107a-IFNg-IL2-TNFa-
		4cytokine			4cytokine			4cytokine
		3cytokine			3cytokine			3cytokine
		2cytokine			2cytokine			2cytokine
		1cytokine			1cytokine			1cytokine
		0cytokine			0cytokine			0cytokine
	CD8+	CD107a+		CD8+	CD107a+		CD8+	CD107a+
		IFNg+			IFNg+			IFNg+
		IL2+			IL2+			IL2+
		TNFa+			TNFa+			TNFa+
		CD107a-			CD107a-			CD107a-
		IFNg-			IFNg-			IFNg-
		IL2-			IL2-			IL2-
		TNFa-			TNFa-			TNFa-
		CD107a+IFNg+IL2+TNFa+			CD107a+IFNg+IL2+TNFa+			CD107a+IFNg+IL2+TNFa+
		CD107a+IFNg+IL2+TNFa-			CD107a+IFNg+IL2+TNFa-			CD107a+IFNg+IL2+TNFa-
		CD107a+IFNg+IL2-TNFa+			CD107a+IFNg+IL2-TNFa+			CD107a+IFNg+IL2-TNFa+
		CD107a+IFNg+IL2-TNFa-			CD107a+IFNg+IL2-TNFa-			CD107a+IFNg+IL2-TNFa-
		CD107a+IFNg-IL2+TNFa+			CD107a+IFNg-IL2+TNFa+			CD107a+IFNg-IL2+TNFa+
		CD107a+IFNg-IL2+TNFa-			CD107a+IFNg-IL2+TNFa-			CD107a+IFNg-IL2+TNFa-
		CD107a+IFNg-IL2-TNFa+			CD107a+IFNg-IL2-TNFa+			CD107a+IFNg-IL2-TNFa+
		CD107a+IFNg-IL2-TNFa-			CD107a+IFNg-IL2-TNFa-			CD107a+IFNg-IL2-TNFa-
		CD107a-IFNg+IL2+TNFa+			CD107a-IFNg+IL2+TNFa+			CD107a-IFNg+IL2+TNFa+
		CD107a-IFNg+IL2+TNFa-			CD107a-IFNg+IL2+TNFa-			CD107a-IFNg+IL2+TNFa-
		CD107a-IFNg+IL2-TNFa+			CD107a-IFNg+IL2-TNFa+			CD107a-IFNg+IL2-TNFa+
		CD107a-IFNg+IL2-TNFa-			CD107a-IFNg+IL2-TNFa-			CD107a-IFNg+IL2-TNFa-
		CD107a-IFNg-IL2+TNFa+			CD107a-IFNg-IL2+TNFa+			CD107a-IFNg-IL2+TNFa+
		CD107a-IFNg-IL2+TNFa-			CD107a-IFNg-IL2+TNFa-			CD107a-IFNg-IL2+TNFa-
		CD107a-IFNg-IL2-TNFa+			CD107a-IFNg-IL2-TNFa+			CD107a-IFNg-IL2-TNFa+
		CD107a-IFNg-IL2-TNFa-			CD107a-IFNg-IL2-TNFa-			CD107a-IFNg-IL2-TNFa-
		4cytokine			4cytokine			4cytokine
		3cytokine			3cytokine			3cytokine
		2cytokine			2cytokine			2cytokine
		1cytokine			1cytokine			1cytokine
		0cytokine			0cytokine			0cytokine

Supplementary Table S4

NK subpopulation and NKT cell phenotype and funcionality (132 variables)

culture	parent population	Variable	culture	parent population	Variable	culture	parent population	Variable
NS	Singlets	Lymphocytes	K562	Singlets	Lymphocytes	PMA + Ionomycyn	Singlets	Lymphocytes
	Lymphocytes	Live CD3-		Lymphocytes	Live CD3-		Lymphocytes	Live CD3-
	Live cells	CD3+		Live cells	CD3+		Live cells	CD3+
		CD3+ CD56+ (NKT)			CD3+ CD56+ (NKT)			CD3+ CD56+ (NKT)
	Live CD3-	CD56+ (Nktot)		Live CD3-	CD56+ (Nktot)		Live CD3-	CD56+ (Nktot)
		CD56hi CD16-			CD56hi CD16-			CD56hi CD16-
		CD56hi CD16+			CD56hi CD16+			CD56hi CD16+
		CD56dim CD16+			CD56dim CD16+			CD56dim CD16+
		CD56dim CD16-			CD56dim CD16-			CD56dim CD16-
	CD3+	CD107+IFNg+		CD3+	CD107+IFNg+		CD3+	CD107+IFNg+
		CD107+IFNg+			CD107+IFNg+			CD107+IFNg+
		IFNg+			IFNg+			IFNg+
		CD107tot+			CD107tot+			CD107tot+
		IFNgtot+			IFNgtot+			IFNgtot+
	CD3+ CD56+ (NKT)	CD107+IFNg+		CD3+ CD56+ (NKT)	CD107+IFNg+		CD3+ CD56+ (NKT)	CD107+IFNg+
		CD107+IFNg+			CD107+IFNg+			CD107+IFNg+
		IFNg+			IFNg+			IFNg+
		CD107tot+			CD107tot+			CD107tot+
		IFNgtot+			IFNgtot+			IFNgtot+
	CD56+ (Nktot)	CD107+IFNg+		CD56+ (Nktot)	CD107+IFNg+		CD56+ (Nktot)	CD107+IFNg+
		CD107+IFNg+			CD107+IFNg+			CD107+IFNg+
		IFNg+			IFNg+			IFNg+
		CD107tot+			CD107tot+			CD107tot+
		IFNgtot+			IFNgtot+			IFNgtot+
	CD56hi CD16-	CD107+IFNg+		CD56hi CD16-	CD107+IFNg+		CD56hi CD16-	CD107+IFNg+
		CD107+IFNg+			CD107+IFNg+			CD107+IFNg+
		IFNg+			IFNg+			IFNg+
		CD107tot+			CD107tot+			CD107tot+
		IFNgtot+			IFNgtot+			IFNgtot+
	CD56hi CD16+	CD107+IFNg+		CD56hi CD16+	CD107+IFNg+		CD56hi CD16+	CD107+IFNg+
		CD107+IFNg+			CD107+IFNg+			CD107+IFNg+
		IFNg+			IFNg+			IFNg+
		CD107tot+			CD107tot+			CD107tot+
		IFNgtot+			IFNgtot+			IFNgtot+
	CD56dim CD16+	CD107+IFNg+		CD56dim CD16+	CD107+IFNg+		CD56dim CD16+	CD107+IFNg+
		CD107+IFNg+			CD107+IFNg+			CD107+IFNg+
		IFNg+			IFNg+			IFNg+
		CD107tot+			CD107tot+			CD107tot+
		IFNgtot+			IFNgtot+			IFNgtot+
	CD56dim CD16-	CD107+IFNg+		CD56dim CD16-	CD107+IFNg+		CD56dim CD16-	CD107+IFNg+
		CD107+IFNg+			CD107+IFNg+			CD107+IFNg+
		IFNg+			IFNg+			IFNg+
		CD107tot+			CD107tot+			CD107tot+
		IFNgtot+			IFNgtot+			IFNgtot+

Supplementary Table S5: ROC coordinates and cut-off values

Treg

Cutpoint	Sensitivity	1-Specificity
0.80	1.000	1.000
1.00	1.000	0.875
1.24	1.000	0.750
1.40	1.000	0.625
1.41	1.000	0.500
1.47	1.000	0.375
1.54	0.833	0.375
1.59	0.833	0.250
**1.63**	**0.833**	**0.125**
1.68	0.667	0.125
2.19	0.667	0.000
2.33	0.500	0.000
3.16	0.333	0.000
3.20	0.167	0.000

EM $CD3^+$

Cutpoint	Sensitivity	1-Specificity
10.20	1.000	1.000
15.00	1.000	0.929
15.20	1.000	0.857
16.70	1.000	0.786
17.30	1.000	0.714
19.90	0.917	0.714
25.10	0.917	0.643
25.30	0.833	0.643
26.80	0.833	0.571
27.00	0.833	0.500
28.80	0.750	0.500
29.50	0.750	0.429
30.30	0.667	0.429
30.70	0.667	0.357
31.20	0.667	0.286
31.80	0.583	0.286
33.00	0.583	0.214
**34.50**	**0.583**	**0.143**
35.00	0.500	0.143
36.00	0.417	0.143
37.00	0.333	0.071
37.90	0.333	0.000
39.40	0.250	0.000
41.10	0.167	0.000
53.20	0.083	0.000

N $CD4^+$

Cutpoint	Sensitivity	1-Specificity
4.43	1.000	1.000
9.76	1.000	0.917
10.80	1.000	0.833
13.70	1.000	0.750
15.20	1.000	0.667
18.00	0.929	0.667
18.20	0.857	0.667
19.40	0.857	0.583
21.60	0.857	0.500
**22.00**	**0.857**	**0.417**
25.70	0.786	0.417
28.50	0.714	0.417
28.60	0.714	0.333
31.50	0.643	0.333
32.10	0.571	0.333
34.80	0.500	0.250
35.80	0.500	0.167
36.10	0.429	0.167
41.90	0.357	0.167
43.20	0.286	0.167
45.50	0.214	0.167
49.40	0.214	0.083
49.50	0.214	0.000
54.90	0.143	0.000
56.70	0.071	0.000

TD $CD8^{hi}$ $CD4^{low}$

Cutpoint	Sensitivity	1-Specificity
1.66	1.000	1.000
2.15	1.000	0.917
2.20	1.000	0.833
2.26	1.000	0.750
2.45	1.000	0.667
2.66	0.929	0.667
3.79	0.929	0.583
4.36	0.929	0.500
5.13	0.857	0.500
5.40	0.857	0.417
5.53	0.857	0.333
7.05	0.786	0.333
7.56	0.714	0.333
8.49	0.714	0.250
**9.03**	**0.714**	**0.167**
9.63	0.643	0.167
10.50	0.571	0.167
10.70	0.500	0.167
12.00	0.429	0.167
12.30	0.357	0.167
12.80	0.286	0.167
27.50	0.286	0.083
34.30	0.214	0.083
35.20	0.143	0.083
55.00	0.071	0.083
60.90	0.071	0.000

EM $CD8^{low}$ $CD4^{hi}$

Cutpoint	Sensitivity	1-Specificity
22.00	1.000	1.000
22.40	1.000	0.929
25.40	1.000	0.857
34.20	0.917	0.857
37.00	0.917	0.786
37.40	0.917	0.714
40.50	0.917	0.643
40.60	0.917	0.571
41.40	0.833	0.571
43.50	0.833	0.500
43.60	0.833	0.429
45.90	0.833	0.357
46.60	0.750	0.357
49.30	0.667	0.357
52.70	0.667	0.286
54.40	0.667	0.214
**54.60**	**0.667**	**0.143**
59.00	0.583	0.143
64.20	0.500	0.143
65.50	0.417	0.143
65.90	0.333	0.143
68.20	0.333	0.071
76.30	0.333	0.000
78.10	0.250	0.000
83.10	0.167	0.000
84.70	0.083	0.000

CM $CD3^+$ $\gamma\delta$

Cutpoint	Sensitivity	1-Specificity
1.69	1.000	1.000
3.03	1.000	0.889
3.58	1.000	0.778
3.80	1.000	0.667
4.03	1.000	0.556
5.57	0.833	0.556
5.68	0.833	0.444
5.95	0.833	0.333
**6.56**	**0.833**	**0.222**
7.35	0.667	0.222
8.49	0.500	0.222
9.82	0.500	0.111
10.10	0.500	0.000
14.30	0.333	0.000
17.80	0.167	0.000

*Red, optimal cut-off values

Supplementary Table S6: ROC coordinates and cut-off values

CD8$^+$ TNFα^+		
Cutpoint	Sensitivity	1-Specificity
1.59	1.000	1.000
2.44	1.000	0.857
2.75	1.000	0.714
3.05	0.889	0.714
3.45	0.889	0.571
4.74	0.889	0.429
6.20	0.889	0.286
**6.57**	**0.889**	**0.143**
7.32	0.778	0.143
7.40	0.667	0.143
8.04	0.556	0.143
9.85	0.556	0.000
10.20	0.444	0.000
11.70	0.333	0.000
13.80	0.222	0.000
15.50	0.111	0.000

CD8$^+$ 3 cytokines (SEB)		
Cutpoint	Sensitivity	1-Specificity
0.76	1.000	1.000
1.07	1.000	0.800
1.53	0.889	0.800
1.62	0.889	0.600
2.61	0.889	0.400
**3.23**	**0.889**	**0.200**
3.52	0.778	0.200
3.61	0.667	0.200
3.64	0.556	0.200
4.46	0.556	0.000
5.60	0.444	0.000
5.90	0.333	0.000
6.65	0.222	0.000
6.98	0.111	0.000

CD8$^+$ MART-1$^+$ 1 cytokine (MART-		
Cutpoint	Sensitivity	1-Specificity
8.33	1.000	1.000
14.68	1.000	0.889
16.08	1.000	0.778
17.70	1.000	0.667
18.44	1.000	0.556
18.80	1.000	0.444
19.09	0.800	0.444
19.34	0.800	0.333
22.97	0.800	0.222
24.42	0.600	0.222
26.87	0.600	0.111
**34.27**	**0.600**	**0.000**
36.36	0.400	0.000
40.00	0.200	0.000

CD8$^+$ MART-1$^+$ 0 cytokine (MART-		
Cutpoint	Sensitivity	1-Specificity
34.30	1.000	1.000
40.00	1.000	0.800
51.20	1.000	0.600
55.90	0.889	0.600
59.90	0.778	0.600
66.90	0.778	0.400
68.80	0.778	0.200
**72.30**	**0.778**	**0.000**
73.30	0.667	0.000
74.10	0.556	0.000
75.00	0.444	0.000
75.60	0.333	0.000
79.80	0.222	0.000
82.30	0.111	0.000

CD56^{dim} CD16$^-$ (NT)$^-$		
Cutpoint	Sensitivity	1-Specificity
3.41	1.000	1.000
3.51	1.000	0.833
3.76	1.000	0.667
3.81	1.000	0.500
4.06	0.857	0.500
4.55	0.857	0.333
**7.72**	**0.857**	**0.167**
8.96	0.714	0.167
9.29	0.571	0.167
10.58	0.429	0.167
14.97	0.429	0.000
17.30	0.286	0.000
23.02	0.143	0.000

CD56^{hi} CD16$^+$ IFNγ^+ (PI)$^-$		
Cutpoint	Sensitivity	1-Specificity
0.00	1.000	1.000
1.66	1.000	0.600
2.00	1.000	0.400
2.67	0.800	0.400
6.06	0.800	0.200
**9.80**	**0.800**	**0.000**
10.53	0.600	0.000
57.15	0.400	0.000
100.00	0.200	0.000

*Red, optimal cut-off values

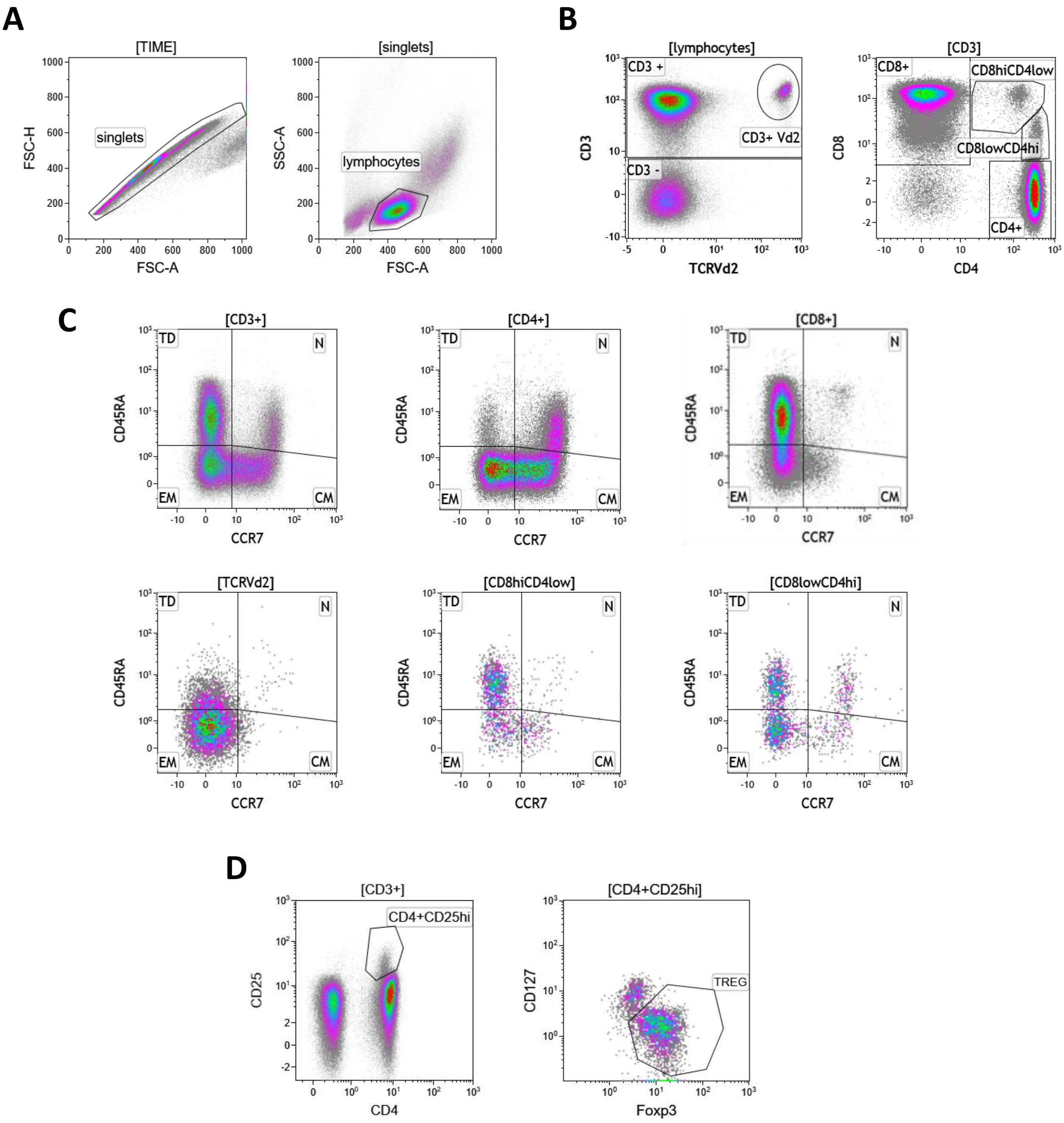

Supplementary Figure S1. Representative dot plots depicting the gating strategies for analyzing different CD3^{+} cell subsets. **A, B and C)** PBMCs were stained with a six-color panel which includes anti-CD45RA, -Vδ2, -CCR7, -CD8, -CD4 and-CD3 mAbs. **A)** Debris and cell aggregates were removed from the analysis by gating on singlets (left plot). Lymphocytes were gated within a SSC-A/FSC-A dot plot (right plot). **B)** A CD3 *vs* TCR Vδ2 dot plot was drawn within the lymphocyte region, allowing the identification of total CD3^{+} T cells, γδ T cells (CD3^{+} TCR Vδ2^{+}) and CD3^{-} cells (left plot). Single positive (either CD4^{+} or CD8^{+} T cells) and double positive CD4^{+}CD8^{+} (either CD8^{hi}CD4^{low} or CD8^{low}CD4^{hi}) T cells were identified within CD3^{+} gated cells (right plot). **C)** The different T cell subsets were then distinguished in naïve (N, CD45RA^{+} CCR7^{+}), central memory (CM, CD45RA^{-}CCR7^{+}), effector memory (EM, CD45RA^{-}CCR7^{-}), and terminally differentiated (TD, CD45RA^{+}CCR7^{-}) cells. **D)** PBMCs were stained with a six-color panel which includes anti-CD3, -CD4, -CD25, -CD127, -Foxp3 mAbs and LIVE/DEAD™ Fixable Near-IR Stain. Regulatory T cells (Tregs) were defined as CD4^{+}/CD25^{hi} (left plot), CD127^{-}/Foxp3^{+} (right plot), within CD3^{+} -live gated cells.

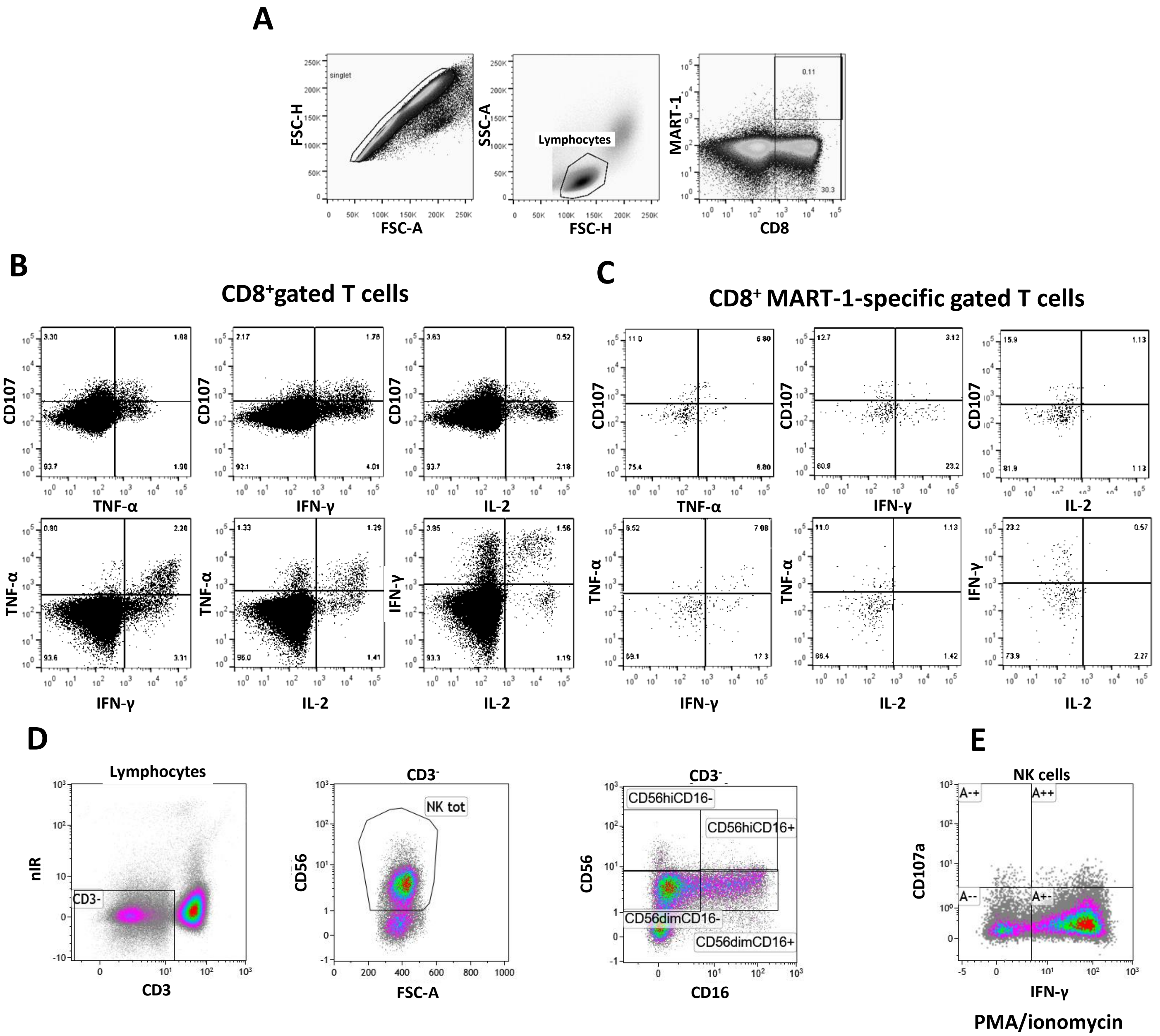

Supplementary Figure S2. Representative dot plots from functional flow cytometry assays. **A, B and C)** PBMCs were stained with a six-color panel which includes anti-CD3, -CD56, -CD16, -IFN-γ, -CD107 mAbs and LIVE/DEAD™ Fixable Near-IR Stain. **A)** Debris and cell aggregates were removed by gating on singlets (left plot). After gating on lymphocytes using a SSC-A/FSC-A dot plot (middle plot), a CD8 *vs* HLA*0201 MART-1 tetramer dot plot was drawn, allowing the identification of total CD8$^+$ T cells and MART-1-specific T cells (right plot). **B and C)** Plots show cytokine (TNF-α, IFN-γ, IL-2) production and CD107a expression of **B)** total CD8$^+$ and **C)** CD8$^+$ MART-1-specific T cells. **D and E)** PBMCs were stained with a six-color panel which includes anti-CD3, -CD56, -CD16, -IFN-γ, -CD107 mAbs and LIVE/DEAD™ Fixable Near-IR Stain. **D)** Total Natural killer (NK) cell subsets were distinguished within the CD3$^-$ region based on the expression of CD56 and further differentiated into four different subsets: CD56^{dim}CD16$^+$, CD56^{hi}CD16$^-$, CD56^{dim}CD16$^-$ and CD56^{hi}CD16$^+$. **E)** Total NK functionality after *in vitro* stimulation with PMA/ionomycin assessed by CD107a and IFN-γ expression.

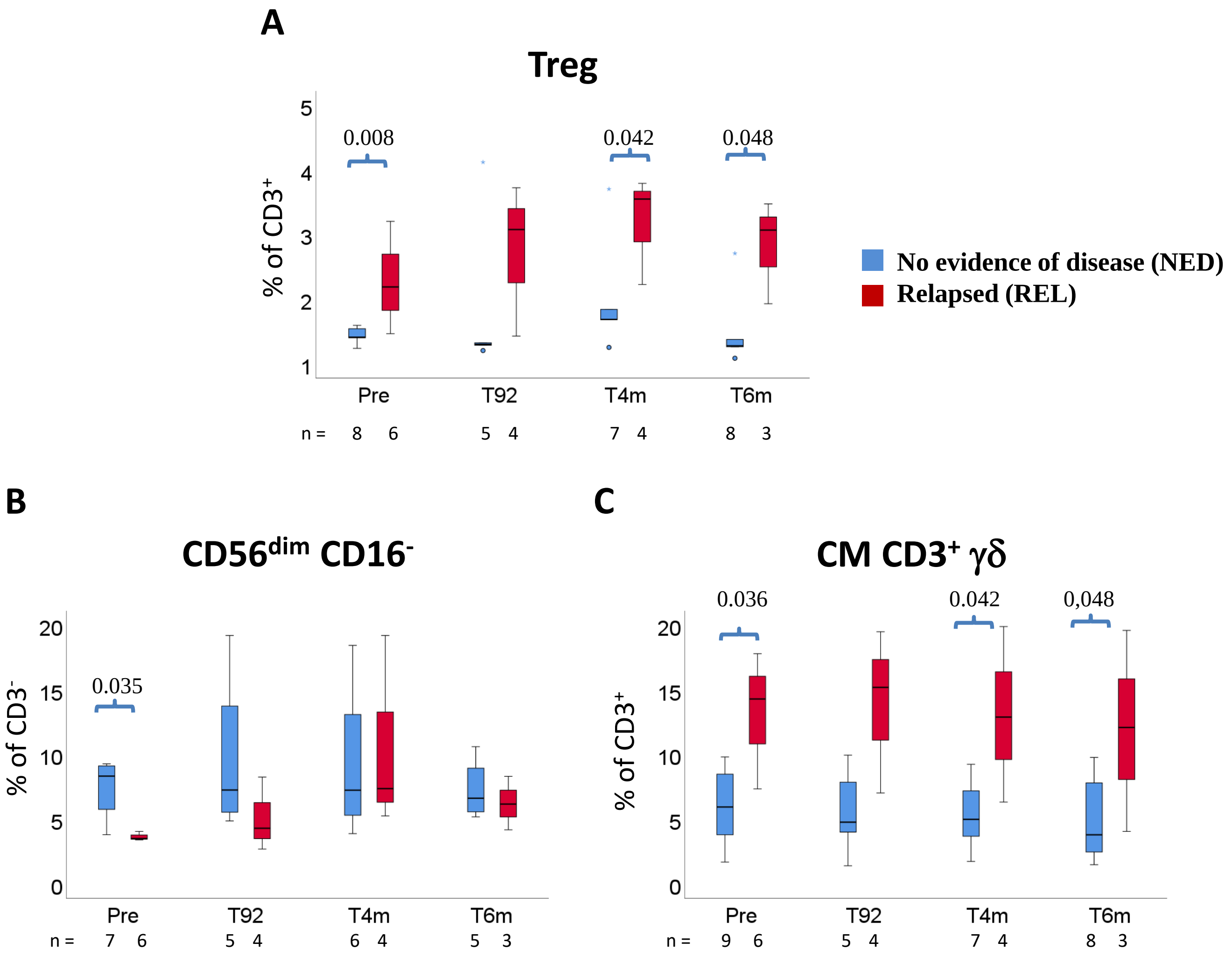

Supplementary Fig. S3. Box plots (showing median, interquartile range, minimum and maximum) representing the frequency of **A)** regulatory T cells (CD3$^+$CD4$^+$CD25^{hi}CD127$^-$Foxp3$^+$) (Treg), **B)** natural killer (NK) cell subset (CD3$^-$CD56^{dim}CD16$^-$) and **C)** central memory (CM) γδ T cells (CD3$^+$TCRVδ2$^+$CD45RA$^-$CCR7$^+$) before (pre), at day 92 (T92) and at month 4 (T4m) or 6 (T6m) following the beginning of treatment, in patients with no evidence of disease (NED) and relapsed (REL). The number of patients (n) is indicated below the graphs. P-values by Mann-Withney non-parametric U test.
